# For reducing premature adult mortality in India, education matters more than income

**DOI:** 10.1073/pnas.2503809123

**Published:** 2026-02-02

**Authors:** Moradhvaj Dhakad, Erich Striessnig, Nandita Saikia, Samir K.C., Wolfgang Lutz

**Affiliations:** ^a^Population and Just Societies, International Institute for Applied Systems Analysis, Laxenburg 2361, Austria; ^b^Laboratory of Demographic Data, Max Planck Institute for Demographic Research, Rostock 18057, Germany; ^c^Department of Demography, University of Vienna, Vienna 1010, Austria; ^d^Wittgenstein Centre for Demography and Global Human Capital (International Institute for Applied Systems Analysis, Austrian Academy of Sciences, University of Vienna), Vienna 1010, Austria; ^e^Department of Public Health and Mortality Studies, International Institute for Population Sciences (Deemed to be University), Mumbai 400 088, India; ^f^Asian Demographic Research Institute, Shanghai University, Shanghai 200444, China

**Keywords:** adult mortality, education, wealth status, multilevel analysis, India

## Abstract

Preventing premature death is a global policy objective reflected in the SDGs. While numerous studies have found socioeconomic factors to be significantly associated with premature death everywhere in the world, the debate on the relative effect of such factors on mid-age (15 to 59 ages) has not received enough attention, particularly in low-income countries, where this question is pertinent due to scant resources in the healthcare sector. Using nationally representative, longitudinal data from the India Human Development Survey, we assess the relative importance of individual- and community-level education vs. income and wealth in India, where approximately 3 million premature deaths occur annually in mid-ages. We find a clear downward gradient in mid-age mortality with increasing education within each wealth category, whereas no consistent mortality advantage with increasing wealth status is visible within education subgroups. Multilevel logistic regression models show that the decline in the risk of death across the education spectrum by far exceeds (14% for male and 13% for female) the decline moving along the wealth distribution, even after controlling for other relevant demographic, socioeconomic, regional variables. Along with the direct effect of individual-level educational attainment, we also find a protective effect of education at the community level, particularly for women. Based on these findings, we infer that educational attainment is essential for reducing mid-age mortality in India. Population and health policies in developing countries, therefore, should focus on education to prevent adult mortality.

## Significance

Due to progress in reducing infant mortality, the global agenda for preventing premature mortality in recent years has been shifting to adults. While there is strong evidence on the role of socioeconomic factors in reducing infant mortality, drivers of adult mortality risk remain underresearched. In this study, we systematically assess the role of education in comparison to wealth in explaining mid-age (15 to 59 y) mortality differentials in India, where one fifth of global adult deaths occur. Our results suggest that both at the individual and community level, the protective effect of education by far exceeds the effect of wealth. This has important implications for the mortality-related SDGs and for setting development priorities in India and other low-income countries.

**Fig. 1.**
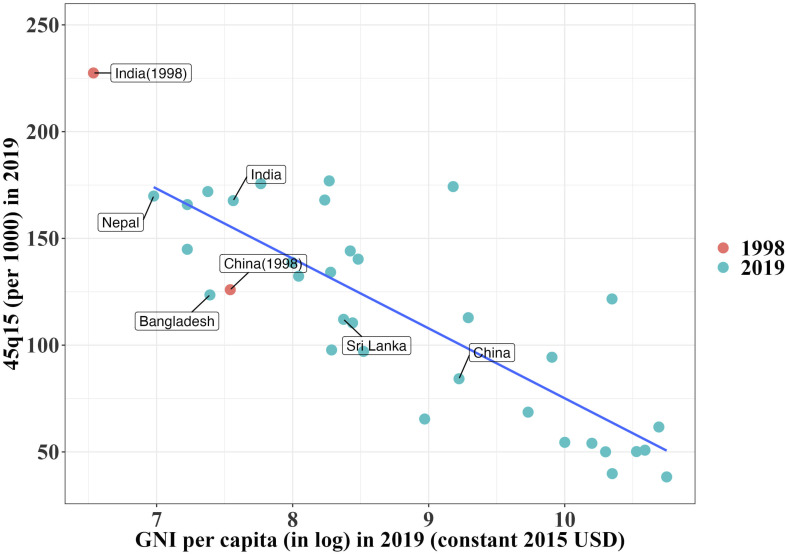
Relationship between real GDP per person (constant USD) and 15 to 59 age-group mortality in the Asian region, 2019. India and China in 1998 added for comparison.

Economic growth is presumed to improve survival conditions and reduce mortality, thanks to the greater availability of resources for investing in social security, public health interventions, and healthcare infrastructures (1, 2). India has long been praised for its solid economic growth. However, not all of India equally benefited from this growth and not all indicators of development confirm the seemingly untarnished success story. Mortality rates, for example, failed to follow trends in economic development (3, 4) and, particularly among Indian mid-age adults (15 to 59 ages), rank among the highest in the world. Fig. 1 shows the correlation between adult mortality in the 15 to 59 age group and economic development across Asian countries. It reveals that India exhibits higher adult mortality rates than neighboring countries like Bangladesh, Sri Lanka. Adult mortality is also higher than its level of economic development might suggest. Notably, China, the other population superpower, has experienced far lower mortality when it reached India’s 2019 level of development back in 1998.

The study of premature mortality among middle-aged individuals in India has global significance for a number of reasons: First, since 2023 India has surpassed China in terms of total population and its mere size makes India the second largest contributor to the overall number of deaths in the world. With about two-thirds of India’s population belonging to the 15 to 59 age group, in 2023 India alone registered one-fifth of the total number of adult deaths worldwide (5). This reflects India’s important role in achieving sizable reductions in global premature mortality as aimed for in the Sustainable Development Goals (SDGs). About half of the 10 million annual deaths in India in 2019 occurred to people under the age of 60, and the share of deaths in the 15 to 59 age group has been consistently increasing in recent decades because of the strong decline in under-five mortality. In 2018, India’s under-five mortality rate was lower than the global average, whereas mortality among adults aged 15 to 59 remained well above the global average (6).

Second, ages 15 to 59 correspond to the official working age in India’s public sector. Premature mortality is a tragedy, no matter if occurring in infancy or at mid-ages. Yet the economic repercussions of an adult dying, possibly at the peak of their productivity and while being one, if not the only breadwinner of the family, are far more impactful. Ample evidence from qualitative research confirms the detrimental consequences that losing a primary wage earner has on the rest of the family, ranging from the survival chances of orphaned children to the disruption in children’s education, early marriage of girl children, reduced income, and consumption expenditure of the bereaved household (7, 8). The loss of an individual’s labor and related income can give rise to volatile consumption patterns and food insecurity (9). Consequently, within the context of the SDGs, high mid-age mortality not only reflects a lack of progress toward SDG-3 (good health and well-being), but it also stands in the way of achieving other goals such as ending poverty (SDG-1), hunger (SDG-2), raising education levels (SDG-4), or achieving gender equality (SDG-5).

Third, India serves as an important case study for understanding adult mortality in Low- and Middle-Income Countries (LMICs) due to its unique combination of demographic and epidemiological transitions. The country is experiencing a rapidly increasing burden of noncommunicable diseases (NCDs), fueled by longer life expectancy and changing lifestyle patterns, which are characteristic of many LMICs undergoing development. Moreover, India’s development has been accompanied by rising mortality from accidents, suicides, and stress-related conditions, such as cardiovascular and digestive diseases (10).This dual burden of NCDs and non-natural causes of death makes India a representative example for other LMICs facing similar transitions. Despite that, rising adult mortality remains a neglected public health issue with a paucity of research in LMICs, often due to incomplete vital registration systems. Analyzing adult mortality trends in India can help to develop preventive strategies not only in India but also in other LMICs.

Finally, India faces stark disparities in mortality due to income inequality, gender, rural–urban divides, and healthcare accessibility. Understanding the relative importance of education and wealth in mortality reduction can help predict future trends within the country. This knowledge will improve global mortality estimates and helps prioritize international public health efforts.

Ever since Kitagawa and Hauser’s (11) pioneering work on socioeconomic differentials in mortality in the United States, numerous studies have documented the role of socioeconomic factors across time and place. Higher socioeconomic status typically results in a reduced risk of premature death (12–14), albeit with great variation by population and over time (15). In addition, some studies from Western countries also examined mortality disparities by religious affiliation, ethnicity, and race (16, 17). Yet since an individual’s affiliation to one of these population subgroups cannot easily be changed, the main focus of social policies has been on facilitating social mobility by raising people’s status in the dimensions of education and income. An important limitation with much of the available research is that socioeconomic factors are often studied either in isolation, resulting in inflated effect sizes and making it hard to assess which factors to prioritize in reducing mortality risk (18, 19), or combined within the concept of socioeconomic status, recognizing the difficulties behind identifying the effects of its integral components (20).

Due to the lack of complete and robust vital registration systems, the scant evidence on socioeconomic mortality differentials in LMICs is primarily based on the study of under-five mortality. These studies are facilitated through Demographic and Health Surveys (DHS) that collect women’s birth histories from many countries (21). This focus of mortality research in LMICs is also a consequence of child mortality still being much higher there, thus relegating mid-age mortality to a subordinate challenge in terms of necessary public health interventions. If taken as an indicator of a population’s well-being, life expectancy does, of course, increase much faster by lowering the risk of dying during early life stages (22). From a policy perspective, though, it is intriguing that the topic of premature mortality at prime working ages has not received more attention, particularly in light of the scarce resources available for healthcare in most of the LMICs.

The present study tries to improve upon this situation by looking into the socioeconomic drivers of mid-age mortality in India. In particular, we compare the effects of educational attainment (no education, primary, lower secondary, upper secondary, postsecondary, and above) and economic status measured by household wealth status (poorest, poor, middle, rich, richest). Using nationally representative longitudinal data from the India Human Development Survey (IHDS) conducted in 2004–05 and 2011–12, we track individuals from survey wave 1 to 2 and relate their risk of dying to their individual socioeconomic characteristics, as well as those of their community, in a multilevel modeling framework. To understand the pathways through which education and wealth are associated with mid-age mortality, we carry out mediation analysis using further socioeconomic factors (work status-type of occupation, caste, affiliation, etc.), health behaviors (alcohol or tobacco consumption), and health status as potential mediators. The goal is to provide crucial information for targeted interventions aimed at reducing premature death at working ages.

## Income vs. Education

The literature knows various channels through which education can affect adult mortality. First of all, education enhances cognitive skills, problem-solving abilities, and learning effectiveness, thus contributing to the build-up and maintenance of human capital (23). Adults with higher levels of education are less likely to engage in risky behaviors, such as smoking and drinking, and are more likely to incur in healthy behaviors related to better diet and exercise (3, 24, 25). Education offers opportunities to learn about health-inducing behaviors, which enables people to recognize symptoms of ill health in a timely manner and seek appropriate medical support (26, 27). Individuals with more education tend to live and work in healthier environments (28–30). Moreover, education affects people’s social networks, as well as the mobility and portability of important social connections, which have been shown to have positive health effects (31–33). Education also reduces the risk of death by enhancing job opportunities. Some empirical research suggests that more educated individuals tend to have longer investment horizons, are more risk-averse, and suffer from mental health problems to a lesser degree (34–36). Education also affects psychosocial factors in health, such as a person’s sense of control, risk of anxiety, depression, social isolation, and stress (37).

Likewise, the link between economic status and health has been widely documented (38–40), with poverty being widely recognized as one of the major determinants of poor health. Individuals with higher income can afford to live in safer houses and neighborhoods, purchase higher quality food, have better access to recreational facilities, and receive better healthcare. Higher economic status comes with greater prestige, which can increase self-esteem and affect health positively. However, increased stress levels among high-earning individuals, linked to fears of losing one’s economic status and not “keeping up with the Joneses” (41), might be related to a higher risk of cardiovascular diseases (CVD) (42). Income levels are found to be strongly associated with mortality risk, controlling for other factors such as disability and bad neighborhood characteristics (43).

Although individual education and income have proven to be strong predictors of morbidity and premature mortality in high-income countries, research based on LMICs as well as global macrolevel studies have been scarce. A recent exception is a study by Lutz and Kebede (19), estimating the relative effects of educational attainment (mean years of schooling) and income (GDP per capita) on life expectancy at birth and child mortality. Their results suggest that education has been more relevant than income in improving mortality conditions across the globe in the past decades. Between these types of studies, limited research focuses on the community-level effects of socioeconomic status. Exceptions are studies by Winkleby and Cubbin (44) as well as Ribeiro et al. (45), both of which find protective effects from better-educated individuals to those with less education through social learning at the neighborhood level. The risk of death declined, not only with the rising education level of the individual but also among low-educated individuals who benefitted from the higher educated in their community.

## Results

**Fig. 2.**
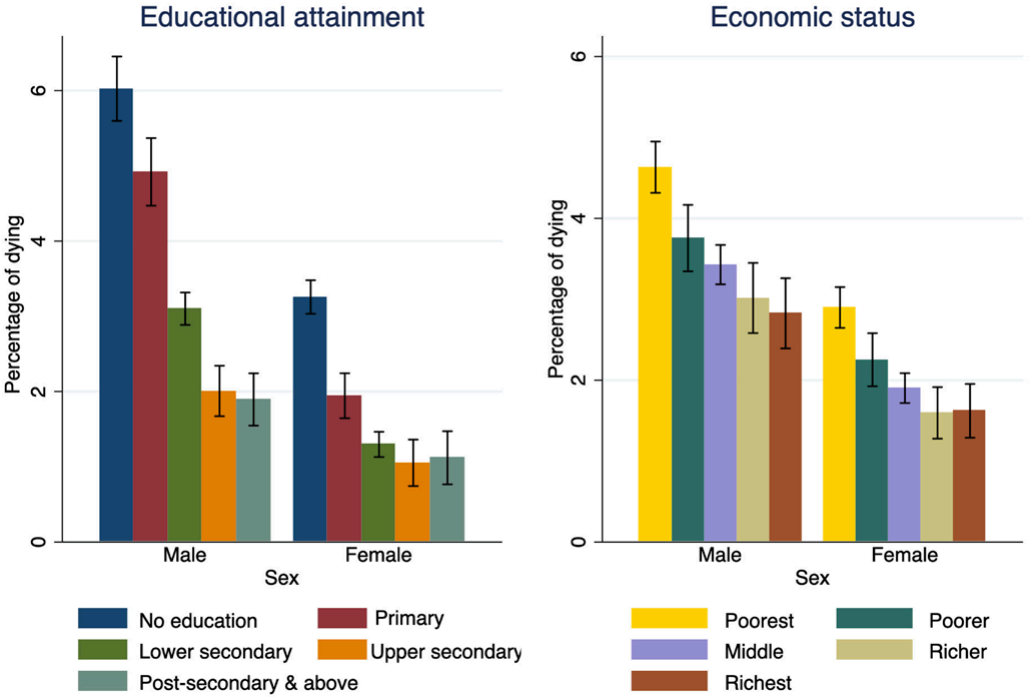
Percentage of men and women dying in mid-age between 2004–2005 and 2011–2012 by educational attainment and economic status (in 2004–2005). Note: Vertical lines indicate 95% CI.

As our main focus is on socioeconomic factors, Fig. 2 shows the mortality patterns of adults dying in mid-age during the 2004–05 to 2011–12 period by educational attainment and household economic status. Additional results by broad age groups are shown in *SI Appendix*, Fig. S4. Of the mid-age adults interviewed in 2004–05, about 3% died before 2011–12 (for a detailed breakdown by other factors see *SI Appendix*, Table S3). When looking at educational attainment (Fig. 2, *Left* side), the percentage of wave-I respondents that died prematurely is about three times higher among women and men without any formal education compared to those with a tertiary degree, with the largest gap opening between those holding less than a secondary degree and those above that threshold. Similarly, we find a significantly higher percentage of deaths among the poorest wealth category than the richest, for both women and men (Fig. 2, *Right* side).

While improving education and economic status both appear to be valid strategies for lowering mid-age mortality in India, under this merely descriptive perspective it remains unclear which one is more effective. To shed more light on the role of educational attainment and economic status in mid-age adult mortality between 2004–05 and 2011–12, we apply multilevel mixed-effect logistic regression differentiating between individual- and community-level effects. While educational attainment and economic status are of course also correlated in India, at only 0.48 this correlation is smaller than in many other places. Considering the gender differences in mortality and causes of death, we run separate regressions for women and men.

**Fig. 3.**
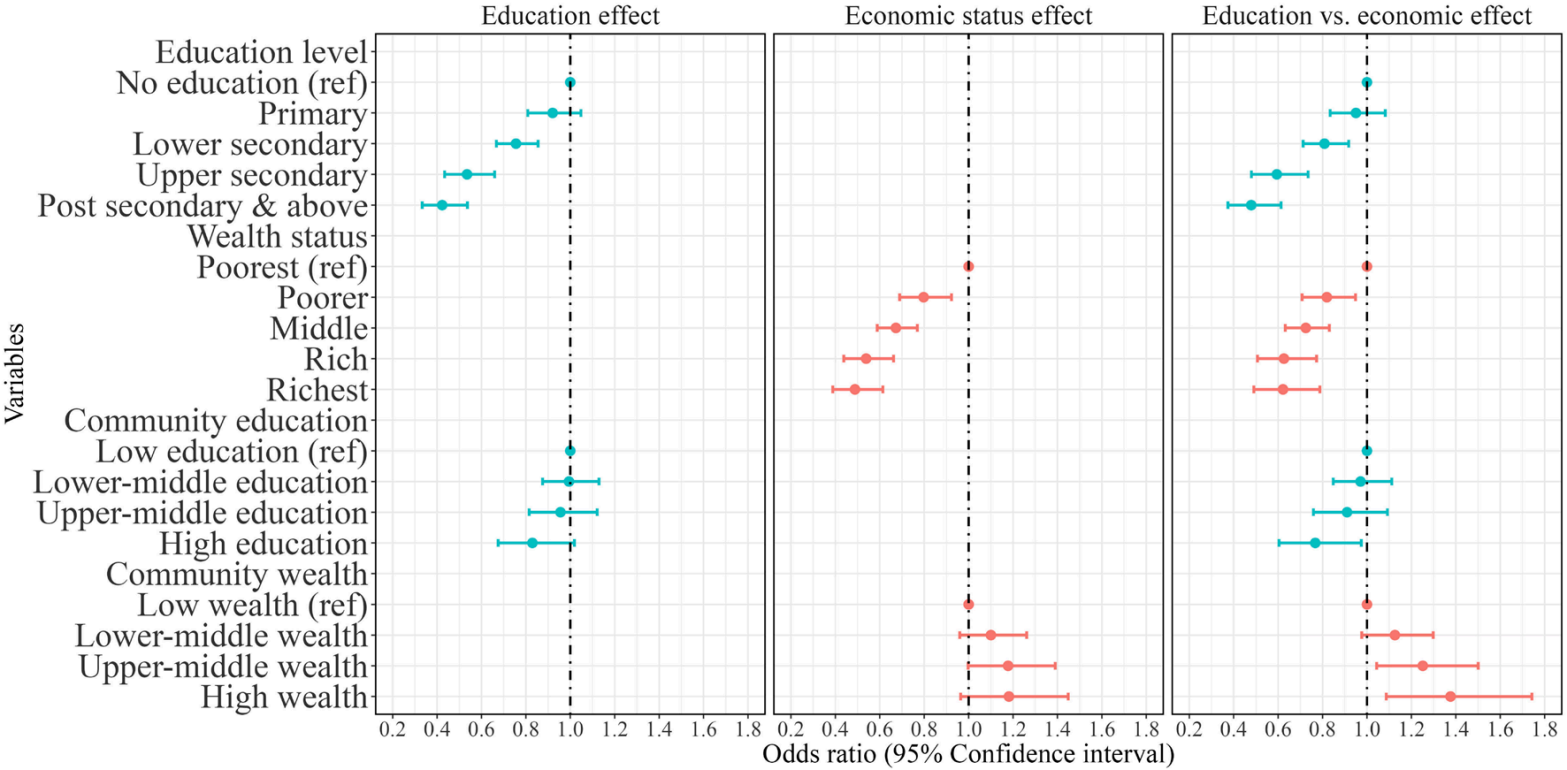
Mid-age mortality between 2004–05 and 2011–12 by education and wealth status for men in India (in 2004–2005). Note: Odds ratios (95% CI) are adjusted for age, health status, marital status, social group, health behaviors (consuming Alcohol/Tobacco), working status and type of occupation place of residence and region.

Fig. 3 shows the odds ratio of dying for men. Panel 1 (*Left* side) and Panel 2 (*Middle*) show the effect of educational attainment and household wealth status, respectively, on male mid-age mortality at individual and community levels. Results in all three panels control for individual-level health status (having any of the following types of morbidity: cataract, tuberculosis, high BP, heart disease, diabetes, asthma, other diseases such as cancer, polio, paralysis, epilepsy, mental illness, STD or AIDS, and any other long-term diseases), demographic characteristics (age and marital status), as well as social affiliation (caste and religion), risky health behaviors (alcohol or tobacco consumption), employment status (economically active and type of employment), and regional characteristics (rural–urban, major region of India). Findings displayed in panels 1 and 2 suggest that the likelihood of death in mid-age decreases significantly with increasing individual-level education and household wealth status. Moreover, community-level education is significantly related to lower mortality risk, however, male mortality in mid-ages appears to be higher in wealthier communities.

Panel 3 on the right-hand side of Fig. 3 shows the independent effects of educational attainment and economic resources measured at the individual and community levels, controlling for demographic, social, health, and regional characteristics. The results suggest that adults’ education levels significantly affect the risk of dying. Compared to those without any education, males with lower secondary education were 20% less likely to die, followed by those with upper secondary and postsecondary and above, who were 41% and 52% less likely to die, respectively. Similarly, the individual’s household wealth status is significantly associated with a reduced risk of death. In comparison to adults residing in the poorest households, the risk of death is 27% lower among those with middle wealth status, followed by those living in the rich (−37%) and richest (−38%) wealth category households.

Taken together, these findings indicate a much steeper decline in mid-age mortality across the education distribution when compared to the decline across equally sized wealth categories. For instance, upper-secondary educated men experience a lower risk of death than men residing in India’s wealthiest household category of roughly the same size. A similar, protective effect of education against premature mortality can be identified at the community level, whereas the comparable community-level wealth index indicates higher, rather than lower risk of dying in wealthier communities, especially for males.

**Fig. 4.**
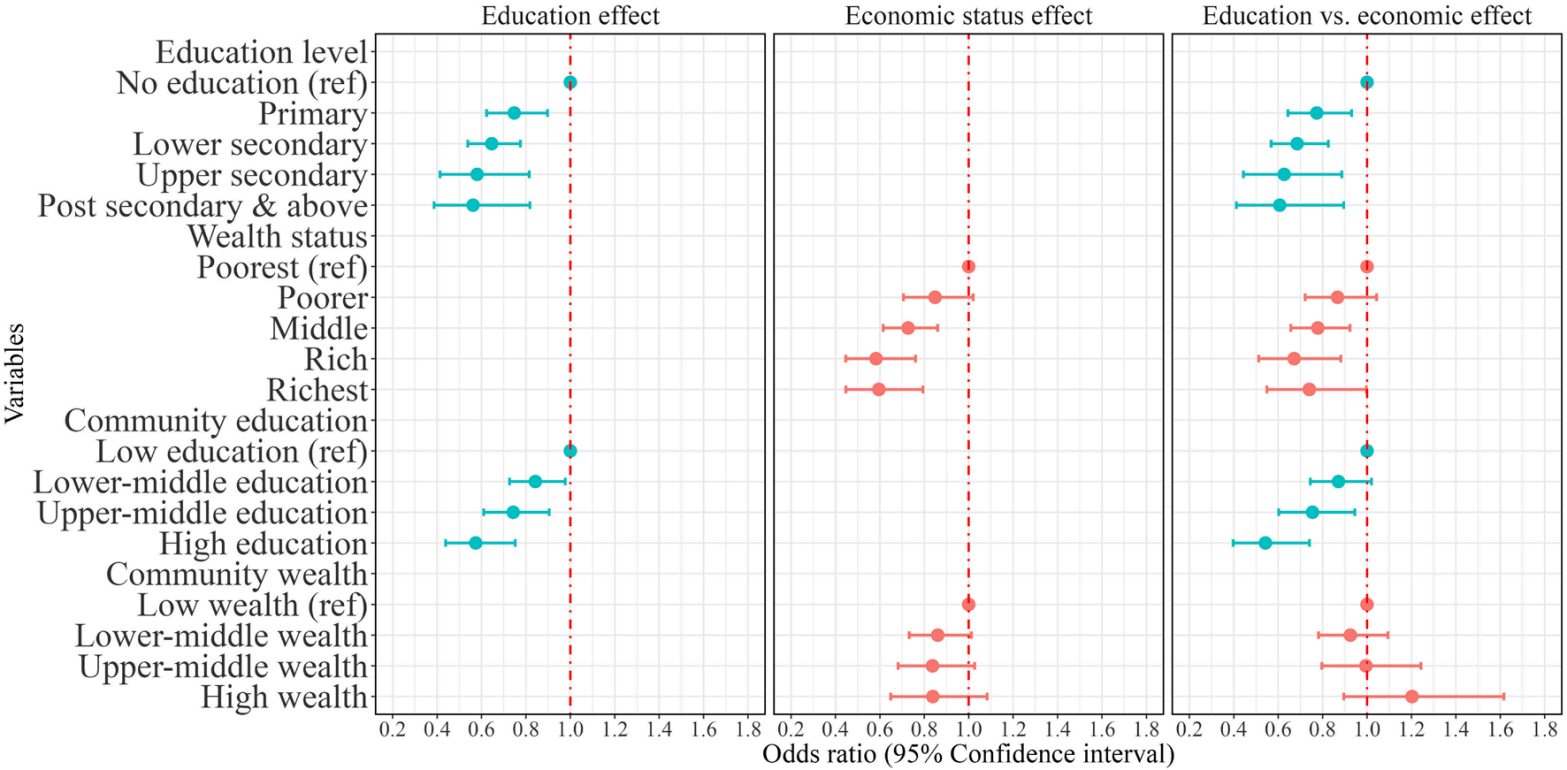
Mid-age mortality between 2004–05 and 2011–12 by education and wealth status for female in India (in 2004–2005). Note: Odds ratios (95% CI) are adjusted for age, health status, marital status, social group, health behaviors (consuming Alcohol/Tobacco), working status and type of occupation place of residence and region

Fig. 4 shows the odds of dying in mid-age for women, controlling for the same factors as before. Both higher levels of education and household wealth status are again related to a substantially reduced likelihood of women dying during their mid-ages. Panel 3 of Fig. 4 shows the net effects of educational attainment and economic resources measured at individual and community levels. Results indicate that even after controlling for economic status and other background characteristics, the impact of education on female mortality, at both the individual and community levels, remains highly significant. Compared to women without any education, primary-educated women experience a 22% lower likelihood of dying. This advantage increases to 32% among those with lower secondary, and 37% and 39% among women with upper secondary and above levels, respectively. Similarly, household wealth status is negatively associated with the risk of dying. Women’s likelihood of dying was significantly lower among women living in the middle (−22%), rich (−32%), and richest (−25%) households compared to those living in the poorest households.

As was the case for men, though, our findings for women indicate a larger protective effect from education than household wealth. The survival advantage of lower secondary educated women compared to women without education is larger in magnitude than the advantage experienced by those living in the richest households compared to those living in the poorest households. Regarding the community-level effects, unlike men, women residing in an upper-middle educated community (with on average 5 to 7 y of schooling) and high educated community (7+ years of schooling) faced 25 and 45% lower risk of death, respectively, than those residing in low educated communities (with on average <3 y of schooling). But the higher community-level wealth index does not appear to be significantly related to a lower risk of dying in the female part of the sample.

Results for other covariates associated with mid-age mortality (e.g., major morbidities, marital status, caste and religious affiliation, health behavior, work status-type of occupation, rural–urban, and regions), for both women and men in India, explained and reported in *SI Appendix*, Tables S4 and S5.

While stock measurement of wealth is generally preferable to potentially more volatile flows, in further robustness checks we also consider the relationship of mid-age mortality with household income and monthly consumption expenditure. The findings confirm our main result based on the stock measurement of household wealth, with the estimated effect of education continuing to be higher (*SI Appendix*, Figs. S7 and S8 and Tables S6–S9).

**Fig. 5.**
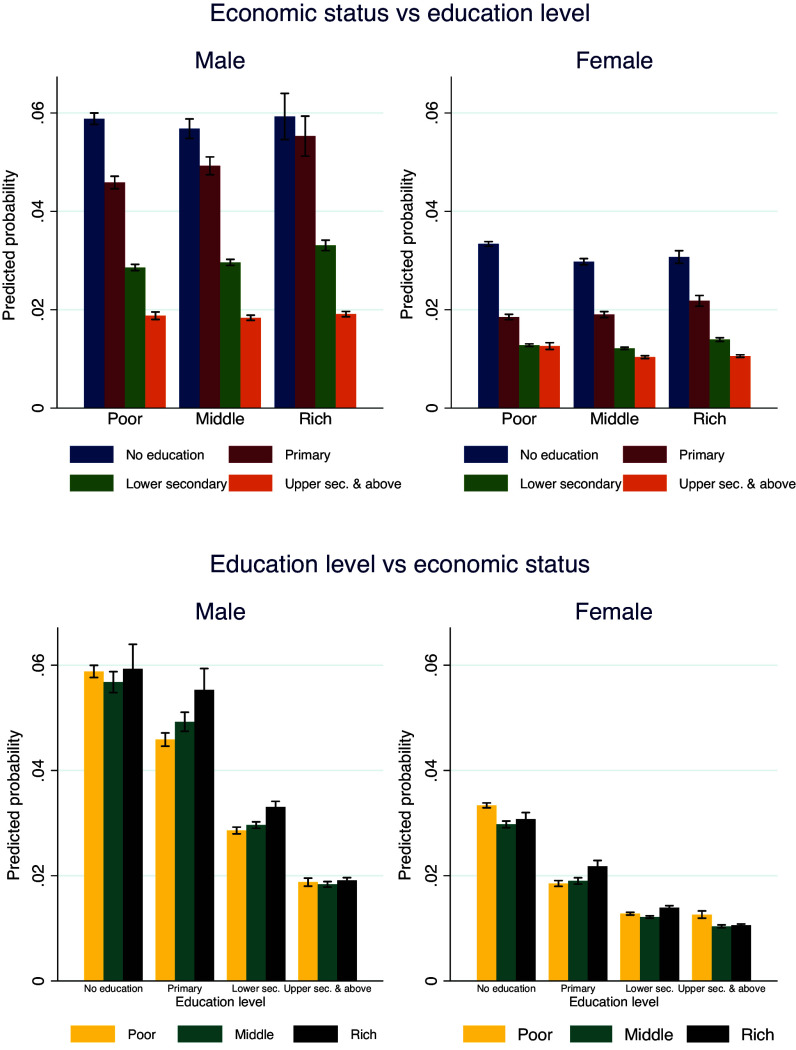
Predicted probabilities of adult deaths in mid-age across economic groups by educational attainment and vice versa. Note: Vertical lines indicate 95% CI.

Fig. 5 shows the predicted risk of dying using the parameter estimates from Panel 3 of Figs. 3 and 4 to understand the effect of educational attainment across wealth categories and vice versa. Increasing education almost uniformly reduces the risk of mid-age death across all wealth categories for both women and men. Irrespective of household wealth status, the risk of mid-age death is reduced by about 65% for both women and men when comparing uneducated individuals to those with upper secondary and above levels of education. Having said that, improving the economic status of households is not predicted to reduce the risk of mid-age death for males across education groups. Rather, the risk of dying during mid-age increases for males from the wealthiest households with less than upper secondary education, while we observe no significant differences in economic status among males with upper secondary and above education levels. In the case of women, the effect of improving household economic status on mid-age mortality does not differ significantly across educational groups.

Finally, to understand what drives the differential effects of education and economic status on mortality, we conduct mediation analyses applying the Karlson-Holm-Breen (KHB) decomposition method (46). This widely used method allows us to partition the overall effects of our main variables of interest into a direct and indirect component, as well as to identify the mediating variables and their respective strength. The mediators we included are health behaviors (alcohol and tobacco consumption), health status (presence of major morbidity), type of occupation, as well as further sociodemographic factors besides education and wealth (caste, religion, marital status).

Consistent with our main findings, the mediation analysis confirms that the remaining direct effect of education (after accounting for mediators) in lowering mid-age mortality, although somewhat attenuated, continues to be far stronger than that of wealth. While in the case of education, only 21% for males and 13% for females are mediated through the included factors, for wealth, these figures amount to 63% for males and 32% for females. This suggests that the pathways through which wealth affects mortality are more dependent on intermediary factors than those for education.

A closer look at the indirect effects shows that education reduces mortality primarily through better occupational outcomes, healthier behaviors, lower major morbidity, and caste affiliation-for both men and women. In contrast, the much larger indirect effect of wealth is largely mediated through caste affiliation, followed by healthy behavior, occupation type, and marital status. Interestingly, for women, occupation does not appear to mediate the relationship between wealth and mortality, likely due to rather low female labor force participation in India. The most striking result of our mediation analysis, though, is the negative indirect effect of wealth on mortality that is mediated by increased morbidity. While education is associated with better health outcomes and thus lowers mortality risk, the opposite seems to be the case for wealth and might, at least partly, explain why education exerts a stronger protective effect against mortality overall (*SI Appendix*, Tables S12 and S13)

In a last step, we also examine the interplay between education and wealth by including both as mediators for the other. When wealth is treated as a mediator for education, it accounts for a small portion of the effect. However, when education is included as a mediator for wealth, it accounts for over 50% of the indirect effect. This further underscores the greater role of education in shaping health outcomes compared to economic status.

## Discussion

Our comparison of the effects of two major aspects of development—educational attainment and economic resources—on mid-age mortality, at first sight suggests that both significantly reduce the risk of dying in mid-age for both women and men. However, upon closer inspection, we find that educational attainment is associated with a much larger decline in mid-age mortality than household wealth status. Men with upper secondary education face a similar risk of premature death as men from the richest households. Similarly, women with lower secondary education are comparable to women from the richest households in terms of their survival chances, and this advantage is growing for women with higher than lower-secondary education.

Our mediation results provide additional insight into why education exerts a stronger protective effect on mid-age mortality than wealth. Higher education is associated with improved health outcomes and reduced mortality risk, while wealth shows a negative association with morbidity, which subsequently elevates mortality risk. There are many potential explanations for these patterns, including differences in lifestyles, access to healthcare, and social determinants of health. Unlike many Western countries, where the evidence on the association between economic status and health is mostly unambiguous, indicating healthier lifestyles and the consumption of more diversified food in response to greater wealth, research from India and other LMICs suggests both positive (47) as well as adverse effects on adult health (48). For instance, CVD and related risk factors, such as diabetes, obesity, and hypertension, are disproportionately prevalent among India’s higher socioeconomic groups, particularly among the richest individuals (49, 50). Wealthy individuals are more likely to engage in nonmanual work with less physical activity, reducing the need for caloric intake (51). Conversely, increasing income can result in a greater than necessary food intake, resulting in obesity, diabetes, and CVD (48, 52), which are more likely to be found among members of the wealthier strata of society (53). The rise in calorie consumption among high-income Indians is a major driving force behind the growing prevalence of diabetes, obesity/overweight, and CVD (54).

Further explanations for the observed trends in mortality among the mid-age group can be found in lower improvements in education that have not kept pace with economic growth (*SI Appendix*, Fig. S3), increased stress levels, unsafe workplace conditions, and the growing number of motorized vehicle injuries. While India’s rapid economic expansion has been linked to a rise in motor vehicle ownership (55), the enforcement of important roadside safety regulations has not kept pace, leading to a significant increase in casualty figures (56). The educational gradient among these is very clear, with up to four times higher risk of death from vehicle crashes among less educated adults compared to the higher educated. However, there appears to be no significant difference in risk by economic status (57, 58). With regard to work-related stress, recent evidence from China suggests that economic growth has been associated with an increase in mental health issues (59). Increased stress can have knock-on effects on other ailments, such as CVD, for instance (60). The financial cost of work-related stress to society has been well documented (61), but more evidence is needed on its mortality impact.

A recent study on the medical impacts of economic development speaks of a “Heart Kuznets Curve” to describe the pattern of elevated blood pressure during earlier stages of development followed by a decline later in the development process (62). Similar patterns are to be found when looking at workplace safety conditions: Studies from more developed economies, where higher levels of GDP are accompanied by greater investment in safer production technologies and improved workplace security protocols, generally show a positive relationship between economic growth and occupational safety (63, 64). However, evidence from Türkiye and Nigeria suggests otherwise, particularly in the short term, when profits are likely to be prioritized over security (65, 66).

Aggregated to the national level, our estimated effects suggest that the unhealthy behavior and lifestyles of less educated rich individuals, particularly men, have been related to higher mid-age mortality in India. Becoming wealthy without increasing human capital does not necessarily benefit adults’ health. Education, therefore, should be viewed as a priority over raising household wealth in India. While it remains to be seen whether our findings have validity for other parts of the developing world, they do confirm earlier findings from more advanced economies, particularly the United States, where increasing economic output by itself has often failed to translate into increasing chances of survival for all (67–69). Rather, overall declines in US life expectancy in the last decade have largely been attributed to an increase in adult mortality among those aged 15 to 59 that have become visible since the 1990s (69). As Lange and Vollmer (70) point out in a recent review, the empirical evidence on the aggregate effect of economic growth on population health is rather mixed and inconclusive. This is in part due to the sector-specific distribution of growth within the economy (71). Growth in sectors that rely more heavily on less skilled labor, such as agriculture, tends to benefit poverty alleviation and resulting improvements in survival. Globalization that enforces specialization can work the opposite way (72).

Our results also suggest the existence of a community-level effect of education for women, but this effect is not stronger for men. While there is considerable heterogeneity across and within Indian communities (73), women residing in, on average, better educated-communities seem to be enjoying a protective effect through their social surroundings. Community-level characteristics can have numerous impacts, including through better access to healthcare and imitation, whereby less-educated women would adopt health-conducive behaviors from better educated women around them (74, 75). A review of community-level interventions to reduce maternal mortality suggests that community-level education is an important factor in reducing female deaths at reproductive ages (76).

Another significant effect with a less straightforward explanation regards the survival disadvantage of mid-age men living in economically better-off communities. On the one hand, there is the aforementioned frontrunner disadvantage in adopting new, potentially unhealthy lifestyles related to disease patterns that were formerly rare in India. On the other hand, disadvantaged individuals might suffer even more from their disadvantage when living inside an otherwise wealthy community. As suggested by recent evidence from the United States on “allostatic load”, indicating cumulative exposure to stress over the life course, the challenge of “Keeping up with the Joneses” is far more challenging for lower status individuals living within otherwise well-off neighborhoods (77), thus raising their stress levels and subsequent risk of premature death. Another potential explanation for individuals with a low socioeconomic profile living in wealthy communities being at greater risk of premature death comes from Taiwan (78), where—despite universal healthcare coverage—lower-status individuals still show worse healthcare utilization and thus face higher risk of dying. Moreover, wealthier communities are often characterized by higher costs of living, which is particularly burdensome for and negatively impacts the health of less wealthy people (79).

Despite several strengths of this study such as the use of nationally representative longitudinal data and the application of a multilevel modeling approach to examine the effects of education and wealth at both individual and community levels, there are some important limitations that warrant caution in interpreting the results. First, although our longitudinal design increases the plausibility of causal claims, it does not entirely eliminate the risk of confounding: In addition to data on several well-known determinants of adult mortality, such as individual dietary patterns, physical activity, lifestyle factors, early childhood experiences, and proximity to healthcare facilities, our study lacks information on still mostly underinvestigated factors, such as personality traits and genetics, that could have a mediating influence on the topic of our research. While there is to date no evidence on these factors from India, the scarce research, mostly from Scandinavian countries, suggests strong educational contributions to reduced adult mortality that are independent from genetic factors (80, 81). The only non-Scandinavian study of which we are aware, Halpern et al. (82), finds a reduced but robust association between education and mortality within a sample of male siblings and twins in the United States. Thus, while in the absence of a more complex study design, based on natural experiments or IV-estimation, confounding can never be ruled out, the existing evidence from richer data sources in developed nations supports our finding of a strong association between education and adult mortality in India.

Second, in examining the impact of economic status on mortality between IHDS I and IHDS II, it is important to acknowledge that individuals’ wealth status may have changed over time (83). Moreover, as most studies rely on longitudinal data, the potential impact of survey attrition has to be acknowledged. This study experienced a 10.5% attrition rate between survey waves, which could potentially influence findings if participants have not gone missing at random. To investigate this possibility, we assessed the quality of our mortality data based on other data. This investigation found the quality of our data to be satisfactory. Still, statistical adjustments were made to account for attrition cases (see Data and Methods). It is also important to note that survey responses were often provided by any adult household member capable of reporting information for all individuals, including those absent. This method may introduce bias in self-reported or proxy-reported data, particularly regarding sensitive variables such as health status, health behaviors (e.g., alcohol and tobacco consumption), employment, and education.

Finally, for lack of detailed income or expenditure data, we use household wealth status as a proxy. This may limit the precision of estimated socioeconomic gradients in adult mortality. Although the association between education and adult survival appears robust, future research incorporating more direct economic measures would help clarify the relative contributions of socioeconomic factors to adult mortality. Moreover, the absence of statistically significant regional differences partly reflects small sample sizes for individual regions and corresponding constraints on statistical power.

## Conclusions

The findings presented in this study suggest that education should be considered as a major policy priority for improving adult health in India and other LMICs. In addition to the direct effect of higher educational attainment for the individual, we find community-level effects of education that can improve the health status, especially of women. The negative community-level wealth effect among men, likely mediated through increased morbidity risk, raises the question of sustainability with regard to India’s rapid economic development when expressed in terms of mortality in mid-age. Our results confirm that improving the economic circumstances of Indians reduces the risk of death; however, without parallel investments in better education, some of that effect will be lost, particularly due to increased mortality at mid-ages. Despite substantial economic growth, improvements in educational attainment in India have not kept pace with expectations based on its level of development (*SI Appendix*, Fig. S3). Therefore, more holistic health policies should also encompass more ambitious education goals to strengthen India’s human resource base. This does not require reinventing the wheel but rather following through with SDG-4, which demands to “ensure inclusive and equitable quality education and promote lifelong learning opportunities for all” for both men and women (84).

## Data and Methods

This study is based on the India Human Development Surveys (IHDS) of 2004–2005 and 2011–2012 conducted by researchers from the University of Maryland, and the Indian National Council of Applied Economic Research (NCAER). IHDS is the first nationwide panel survey conducted in India with a sample size sufficiently large to study rare events like adult death. Due to its longitudinal design, which allows us to connect individual deaths directly to living conditions at the time of the first survey, IHDS provides a perfect opportunity to examine the relative effects of education and household economic status on mid-age mortality.

The IHDS adopts a hierarchical sampling design, with clusters defined as primary sampling units (PSUs): villages or settlements in rural areas and towns or cities in urban areas. Within each PSU, households are selected using stratified random sampling. IHDS interviewed 129,388 individuals aged 15 to 59 y in 2004–05; of those adults, IHDS-II successfully identified 115,781 (90%) and 13,607 (10%) individuals were not identified; these are attrition samples. Out of the successfully identified 115,781 individuals, 3,428 adults had died between the two survey waves. A detailed description of the sample used in this study is provided in the *SI Appendix*, Fig. S1.

The outcome variable is the death of the individuals in mid-age (15 to 59 y) who were interviewed in the 2004–2005 survey and died before the second survey was conducted in 2011–2012. The main predictor variables are educational attainment and economic status at the individual and community levels. A descriptive analysis was conducted to assess the proportion of deaths between 2004–05 and 2011–12 by educational level and economic status. Making use of the hierarchical structure of IHDS, we then employ multilevel mixed-effect logistic regression to study the probability of death between survey waves. In addition to education and economic status, there are many other factors that affect mortality; our models control for demographic factors (age and marital status), social group (caste and religion group), individual-level health status (any of the following types of morbidity: cataract, tuberculosis, high BP, heart disease, diabetes, asthma, other diseases such as cancer, polio, paralysis, epilepsy, mental illness, STD or AIDS, and any other long-term diseases), risky health behaviors (alcohol or tobacco consumption), employment status (economically active and type of employment), and regional characteristics (rural–urban, major region of India). To examine the relation of each individual predictor with mid-age mortality, we first apply univariate models, before going on to the multivariate two-level mixed effect models. Finally, to better understand the differential impact of education and wealth on mortality, we conduct mediation analysis to disentangle direct and indirect mortality effects of socioeconomic development mediated through various intermediary factors. Specifically, we employed the Karlson–Holm–Breen (KHB) decomposition method, which allows us to estimate the total effects of education and economic status on mortality and to partition these effects into direct and indirect components (through mediators), as well as to identify the mediating variables and their respective strength.

Like any long-term follow-up study, we are facing sample attrition. About 10% of wave 1 participants remain unaccounted for at the time of resurveying. To check the consistency of the mid-age death estimates derived from IHDS data, therefore, we compared them with those obtained from India’s official Sample Registration System (SRS) and United Nations mortality estimates for the corresponding period. As shown in *SI Appendix*, Fig. S2, age-specific death rates estimated from the IHDS data are almost identical with the SRS and United Nations mortality estimates. Moreover, the analysis of attrition summarized in *SI Appendix*, Table S1 suggests that individuals are not missing at random from wave 2. Hence, to check for bias in our estimates, we adjusted for attrition using multiple imputation as part of our sensitivity analyses presented in the *SI Appendix*, Table S14-S15. Multiple imputation is essentially an iterative form of stochastic imputation used to handle missing data by creating several plausible datasets in which the missing values are replaced with estimated values. Note that our results are only marginally affected by these adjustments. Odds ratios after adjustment are shown in the *SI Appendix*, Tables S14 and S15.

## Supplementary Material

Appendix 01 (PDF)

## Data Availability

Previously published data were used for this work. Note that all data deposited in a publicly accessible database (and therefore not directly available in the paper or SI) [S. Desai, V. Reeve, and National Council of Applied Economic Research. (2019). Human Development Survey Panel (IHDS, IHDS-II), 2005, 2011–2012. Inter-university Consortium for Political and Social Research. https://doi.org/10.3886/ICPSR37382.v1] (85).
