# Supplementary material for: For reducing premature adult mortality in India, education matters more than income
Source: Proc Natl Acad Sci U S A. 2026 Feb 2;123(6):e2503809123. doi: 10.1073/pnas.2503809123 (PMC12890904; doi:10.1073/pnas.2503809123)
Supplement: Supplementary file 1 — Appendix 01 (PDF) [file pnas.2503809123.sapp.pdf]

Supporting Information for

For reducing premature adult mortality in India, education matters more than income

Moradhvaj Dhakad, Erich Striessnig, Nandita Saikia, Samir KC, Wolfgang Lutz

Corresponding authors:

Moradhvaj Dhakad
Email: dhakad@demogr.mpg.de

Erich Striessnig,
Email: erich.striessnig@univie.ac.at

Wolfgang Lutz
Email: lutz@iiasa.ac.at

This PDF file includes:

- Supporting text
- Figures S1 to S8
- Tables S1 to S15
- SI References

1 Few studies on mid-age (15-59) mortality

This study investigates mid-age (ages 15-59) adult mortality by the socioeconomic characteristics, education, and wealth. The study focuses on India, which has a population of over 1.4 billion, more than Europe and North America combined, and can be seen as an important case study for other Low- and Middle-Income Countries (LMICs). This is because of India's still relatively youthful population – over 60% of the population fall within the 15-59 age group. Yet although mortality in this age bracket continues to be well above the global average (1), very few studies have focused exclusively on mid-age adult mortality and its determinants (2, 3). Some epidemiological studies on India have focused on the role of individual and specific risk factors in health, but the broader contributors to adult mortality in India remain poorly understood despite paramount policy relevancy (4, 5). Most existing research on adult mortality in India describes levels and trends, as well as gender differentials (6–9). The factors leading to high adult mortality have been explored to a lesser extent, particularly compared to those leading to infant and child mortality.

In terms of causes of death, it is important to note that adult mortality in India is largely driven by non-communicable diseases and lifestyle factors, such as poor health-seeking behaviors and widespread, unregulated occupational hazards. These factors differ significantly from the determinants of child and adolescent mortality (10). The main reason for the lack of research on adult mortality is a lack of data: While data on child mortality, including socioeconomic characteristics of mothers, are routinely collected in Demographic and Health Surveys (DHS), similar data for adults remains mostly missing across less-developed countries. Therefore, the India Human Development Survey (IHDS), thanks to its longitudinal nature, offers a rather unique opportunity to study the relative effects of educational attainment and economic status on mid-age deaths.

2 Data and Methods

2.1 Data

The research presented here is based on data from the India Human Development Survey (IHDS) waves 2004–2005 and 2011–2012 (11). In IHDS-I (2004-05), 41,554 households were surveyed across 1,503 villages and 971 urban neighborhoods all over India. IHDS-I is a nationally representative survey collecting socioeconomic and health data on over 215,754 individuals from 33 Indian states and union territories. The second round of the survey (IHDS-II) successfully re-interviewed 40,018 the same households from IHDS-I, as well as households that had split off from the original households but continued to reside in the same locality. For each of the original household members in 2004-05, a tracking sheet had to be filled in order to identify current whereabouts and survival status. For those individuals who had changed residency, household members still residing in their original (i.e., IHDS-I) home were asked to provide information on their current occupation, marital and survival status. If the entire household had migrated or died, interviewers collected demographic characteristics and survival status from their closest relatives or friends, as identified by household members in IHDS-I.

In IHDS-I, demographic information for all household members was collected in the Household Questionnaire under the Household Roster section. For each individual listed in the roster, the questionnaire recorded basic demographic characteristics, including sex (Question 2.3: "Sex") and age (Question 2.5: "How old is he/she?").

In IHDS-II, information on household members from the 2005 interview who had subsequently died was collected using a dedicated Tracking Sheet. Specifically, Section 2 of the tracking sheet ("Tracking sheet for household members from the 2005 interview who have died") documented mortality-related information. This section included Question 2.2, which asked: "How many years ago did he/she die?" The response to this question was used to determine age at death for deceased individual.

Fig. S1 provides a schematic depiction of the survey design, as well as the number of individuals relevant to our study. Since we are interested in mid-age death, we restrict our sample to individuals aged 15-59 years in 2004-05. IHDS-I collected information for 215,754 individuals, 129,388 of whom were aged 15–59 at the time of the first survey wave. Of those 129,388 adults, IHDS-II successfully identified 115,781 (90%). 13,607 individuals were not identified, corresponding to survey attrition of somewhat more than 10% (see SI Section 2.2.2 for how we are dealing with that). Out of these successfully identified 115,781 individuals, 3,428 adults had died between the two survey waves.

2.1.1 Mortality estimates from IHDS data and comparison with SRS and United Nations estimates.

In order to evaluate the quality of mortality data in IHDS, first, age-specific death rates (ASDRs) obtained from IHDS are compared with India's official Sample Registration System (SRS) and United Nations (UN) (12) for the corresponding period (2004 to 2012). It is worth mentioning that IHDS and SRS are two separate datasets with differences in their sampling designs. Fig. S2 shows the comparison of IHDS with SRS and UN mortality rates for the same periods. The concordance between the three sources of information on mortality is very high, confirming the reliability of the IHDS-based mortality estimates.

2.2 Method

IHDS used a hierarchical sampling design to select clusters from rural and urban areas as primary sampling units (PSU). Households within PSU were identified using stratified random sampling. In our multilevel setup, we make use of the hierarchical structure of IHDS. We employ multilevel mixed effect logistic regression to estimate the probability of dying in mid-age by educational attainment and wealth status for individuals who were interviewed in the 2004–2005 survey.

Further, to better understand the differential impact of education attainment and wealth status on mortality, we conducted a mediation analysis to disentangle the direct and indirect effects of these factors operating through various intermediary factors. "We used the Karlson–Holm–Breen (KHB) method, which is specifically designed for mediation analysis in non-linear probability models such as logistic regression, thereby addressing the rescaling problem that arises when comparing coefficients across nested models. It allows for the decomposition of the total effect of an independent variable (e.g., educational attainment, economic status) on mortality into direct effects (net of mediators [the effect of education or wealth on mortality after accounting for the mediators]) and indirect effects (operating through mediating variables). In addition, the method enables the identification of the relative contribution of individual mediators to the overall indirect effect, thereby offering insights into the pathways through which socioeconomic factors influence

mortality. The mediators we included are health status (presence of major morbidity), health behaviors (concerning alcohol and tobacco consumption), type of occupation, and socio-demographic factors (caste, religion, marital status).

2.2.1 Individual- and community-level variables

To assess the relative effect of educational attainment and economic status on mid-age mortality, we rely on measurements from both the individual- and community-levels. IHDS provides information on the educational attainment of individuals, which are divided into five categories: No education, primary, lower secondary, upper secondary, post-secondary & above.

Educational information was collected for all household members through the Household Questionnaire, Section 10 (Education: All Household Members). Educational attainment was recorded using Question 10.5: “How many standard years has he/she completed? Responses to this question were used to assign individuals to the five educational categories described above.

The economic status of the household is measured by a standard wealth status, which has been widely used before (13, 14). This wealth status is based a set of 23 variables that measure household possession of basic consumer durables and assets (primarily furniture, electrical devices and appliances, vehicles, etc.), which can be seen as strong predictors of household-level wealth. A household that disposes of all 23 listed items can obtain a maximum score of 23.

Information on household asset ownership was collected through the Household Questionnaire, Section 13 (Ownership of Consumer Goods). In this section, respondents were asked whether the household owned specific assets, with questions formatted in a yes/no manner (e.g., Question 13.2: “Do you own a cycle/bicycle?”). Similar questions were asked for the remaining consumer durables and assets included in the wealth index. Each asset is coded as a binary indicator (owned = 1, not owned = 0)

A household that does not dispose of any assets is assigned an index value of 0. Based on the index, household wealth status categorized into five categories: poorest, poor, middle, rich, richest. Note that for better comparability, wealth categories have been generated to match the size of the education categories.

As a sensitivity analysis, we have also generated an asset-based score using a principal component analysis (PCA) of the various household assets following Barik, Desai & Vanneman, (15). The correlation between the simple summative index and the more complex PCA-based index was 0.99 (15). Therefore, the presented results are based on the summative index.

In addition to wealth status as an indicator of economic status, we examined the effects of two alternative economic measures: household annual income (in Indian rupees [INR]: poorest [<22,350], poor [22,350-<31,690], middle [31,690-<90,000], rich [90,000-<141,000], and richest [141,000 and above]) and household per capita monthly consumption expenditure (in Indian rupees (INR): poorest [0-<477], poor [477-<602], middle [603-<1197], rich [1197-<1743], and richest [1743 and above]. Both variables were categorized into five groups having similar group sizes of the education categories

In addition to the individual-level effects of socioeconomic status, studies have found important community-level effects on mortality, albeit focusing on child health outcomes (16, 17). Data on

adult mortality, however, are more difficult to obtain, particularly with information on individual- and household-level characteristics. In this study, community-level education was derived as the average years of education attained by adults in the clusters or communities. Community-level education is categorized into four groups: low (<3 years of schooling), lower-middle (3-<5 years of schooling), upper-middle (5-<7 years of schooling), high (7 and above years of schooling). Similarly, the community-level wealth index was created from the mean values of the household wealth index values in the various survey clusters. Community-level average index was again categorized into four groups: low (<4), lower-middle (4-<6), upper-middle (6-<8), high (8 & above). Additional community-level economic indicators were derived from household income and per capita monthly consumption expenditure (MCE). The mean household income at the community level was classified (in Indian rupees [INR]) as low (<28,603), lower-middle (28,603-<59,633), upper-middle (59,633-<91,861), and high (≥91,861). Likewise, the mean per capita MCE within each community was categorized (in Indian rupees [INR]) as low (<560), lower-middle (560-<893), upper-middle (893-<1230), and high (≥1230). Similar to individual level variables, the community level education and economic status categories have been generated to closely match the size of each other variable categories.

To control for pre-existing morbidity, we used information on conditions reported by adults in the 2004-05 baseline survey. These conditions included cataract, tuberculosis, high BP, heart disease, diabetes, asthma, other diseases such as cancer, polio, paralysis, epilepsy, mental illness, STD or AIDS, and any other long-term diseases). Demographic controls included age (in years), sex (men, women), and marital status (married, unmarried, widowed, separated). Moreover, we controlled for social groups as defined based on affiliation to a particular caste and religious groups. The traditional all-Hindu caste or *Jati* categorizes into five *Varnas* (translated into English as castes): *Brahmins* (priests), *Kshatriyas* (warriors, royalty), *Vaisyas* (traders and merchants), and the *Sudras* (manual job) and *Dalits* (the untouchables, doing lowest of the menial jobs). There are several sub-castes within these five castes. However, for administrative purpose, the constitution of India classifies traditional caste groups into four broad categories: scheduled castes (SCs), scheduled tribes (STs), other backward castes (OBCs) and general castes (non-disadvantaged castes). The SCs and STs are officially recognized as socially disadvantaged groups. OBCs are another Indian population group recognized as socially- and educationally-backward, but OBCs have a higher status than SCs and STs. Religion categorized into three categories: Hindu, Muslim and Others. We also controlled for the risky health behaviors (alcohol or tobacco consumption), employment status (economically active and type of employment). IHDS collected information about different 85 types of occupation. These are further aggregated into five, broad categories based on the National Classification of Occupations as adopted by the Office of the Registrar General and Census Commissioner for India. Working status and Occupation are summarized in six categories: (1.) Not working, (2.) Professional/admin/manager and related jobs, (3.) Clerical/sales/service-related jobs, (4.) Farmers/agriculture, (5.) Production and (6.) Working but type of occupation not reported. Model also controlled for region variables: place of residence (rural and urban settlement) and broader regions, categorizing India into five distinct regions (North, West, Central, South, East & Northeast). Table S2 Percentage distribution of the sample by demographic, socio-economic, health and regional characteristics.

Information on health conditions, social group affiliation, health behaviors, and economic activity was obtained from the Household Questionnaire and the Education and Health (Women's) Questionnaire.

Pre-existing morbidity was measured using Questions 9.2–9.15, which asked whether a doctor had ever diagnosed any household member with specific conditions, including cataract, tuberculosis, hypertension, heart disease, and other chronic illnesses.

Caste affiliation was recorded using Question 1.13, which asked: “Is the household Brahmin, OBC, SC, ST, or Other?” Religion was obtained from Question 1.14, which recorded the religious affiliation of the household head (e.g., Hindu, Muslim, or other religions).

Risky health behaviors were measured using Questions 12.2–12.4, which asked whether any household member consumed tobacco or alcohol.

Type of employment was captured using Question WS3A (Occupation Code) and the question What kind of work does name do? This information was then used to derive occupational categories as described above.

2.2.2 Adjustment for attrition

As usual with long-term follow-up studies, a major challenge to our analysis came from the sample attrition of roughly 10%. The analysis of attrition (Table S1) shows that the patterns of lost samples are disproportionately concentrated in more affluent households living in urban areas, that are higher-educated and living in rented houses (no homeownership). All of these characteristics are also related to higher likelihood of migration, which is typically the reason for sample losses, together with the splitting of households.

Although the mortality estimates from the IHDS data concur with the official Indian and UN estimates, to avoid possible bias in our estimates resulting from systematic missingness, we adjusted our sample for attrition. Various procedures have been suggested in the literature for dealing with attrition. Table S1 shows that cases are missing at random (MAR), meaning that the missing data is correlated with other observed variables but not with missing. This means that missing values are described by the other observed variable. When data is MAR, multiple imputation is a highly effective method for handling the missing values (18). Multiple imputation is essentially an iterative form of stochastic imputation used to handle missing data by creating several plausible, hypothetical datasets in which the missing values are replaced with estimated values efficiently (19–23). In our case, the missing information is the binary survival status (yes or no) and we are using a predictive, logistic regression model based on the observed data. The variables used as predictors include age, health status, marriage education, economic status and household ownership status. These variables have been found to improve the quality of imputed values in other applications (24, 25).

3 Results

Table S3 shows the survival status of adults surveyed in 2004-05 by 2011-12 grouped by demographic, health and socioeconomic characteristics in 2004-05. The percentages indicate strong variation in mid-age mortality across those different sub-groups. Around 3% of adults died between survey waves; this percentage is higher for men (3.7%) compared to women (2.38%). As expected, the percentage of respondents not surviving to survey time two is increasing with age.

Looking at the distribution by level of educational attainment, we find a higher share of people dying prematurely among adults without any formal education compared to educated adults. Besides that, percentages decline with level of educational attainment. Among those with post-

secondary education, the percentage of adults dying is about three times lower compared to uneducated adults. Similarly, we find smaller percentages of mid-age deaths among adults living in on average better educated communities.

By economic status, mortality appears to be higher among economically disadvantaged compared to the economically better-off adults. As in the case of education, there seems to be a negative association between wealth and the proportion of deaths observed across all age groups (see Fig. S4). Those reported pre-existing morbidity reported higher percentage of deaths than those did not have any morbidity. Similarly, the individual adopted risky health behaviours consuming alcohol or tobacco reported higher deaths.

By social group, we find the highest percentages of deaths among the SCs/STs population, followed by OBCs and Other caste groups. By religion, Muslims have a lower percentage of deaths than Hindus and other religious groups. The percentages of adults dying were higher in rural than in urban areas.

Fig. S5 presents the percentage of deaths disaggregated by age group, sex and marital status. Clear differences emerge across marital categories. For males, mortality is consistently lowest among the married across all age groups, while substantially higher risks are observed among the widowed and separated, particularly in the 45–59 age group where over one-fifth report death. Unmarried males also experience elevated mortality compared to their married counterparts, though generally lower than that of the widowed and separated. Among females, the pattern is similar, with married women showing the lowest mortality. The widowed and separated women display considerably higher mortality, especially at older ages, while unmarried women in the 30–44 age group also experience relatively elevated risks. Overall, Fig. S5 highlights a pronounced survival advantage for the married, with disadvantaged outcomes for the widowed and separated, particularly in later ages. The gender comparison shows that while both men and women benefit from marriage, the mortality penalty associated with widowhood and separation is especially marked among men in the 45–59 age group.

We examined mid-age mortality by educational attainment across economic groups and, conversely, by economic status within educational categories (Fig. S6). The right panel of Fig. S6 indicates a strong and consistent educational gradient: mortality declines steadily with increasing levels of education across all economic groups. By contrast, the left panel shows that improvements in economic status do not yield comparably consistent reductions in mortality within each educational category. In other words, while higher education is uniformly associated with lower mid-age mortality regardless of economic background, higher economic status alone does not consistently translate into mortality reductions across different education groups.

Results from multilevel analysis

Two-level mixed effect logistic regression was used to predict the role of educational attainment and wealth status measured at the individual- and community-level in mid-age mortality between 2004-05 and 2011-12 separately for men and women. Table S4 shows the likelihood of death for men. First, we estimated bivariate regression models to examine the unadjusted association of each explanatory variable with mid-age mortality. We then specified multivariate models to assess the effects of education and wealth. Model 1 evaluated the effect of education, while Model 2 focused on economic status. Finally, Model 3 compared the relative effect of education and economic status to mid-age mortality. All multivariate models controlled for a comprehensive

set of covariates, including health status (presence of morbidity such as cataract, tuberculosis, high blood pressure, heart disease, diabetes, asthma, cancer, polio, paralysis, epilepsy, mental illness, sexually transmitted diseases including HIV/AIDS, or any other long-term condition), demographic characteristics (age and marital status), social group (caste and religion), risky health behaviours (alcohol and tobacco consumption), employment status (economic activity and type of employment), and regional factors (rural–urban residence and major region of India).

The bivariate column shows the relationship between each explanatory variable and the risk of mid-age adult death, adjusting only for unexplained variation at the individual- and community-levels. According to this, the risk of adult death shows a steep increase at higher ages. The risk of premature death is higher among the widowed and separated men compared to currently married men. Compared to uneducated men, the likelihood of mid-age death decreases by 21% for those with up to primary school education, 52% for the secondary educated, 69% for upper secondary, and 71% for post-secondary educated.

Similarly, wealth status is associated with significantly reduced odds of dying prematurely. Compared to people from the poorest wealth group, the odds of dying are 28% lower among the middle and 41% lower among the richest group, respectively. The unadjusted relationship between both education levels and economic status on the one hand and the likelihood of mid-age death on the other is also apparent when looking at the community-level estimates. Men residing in communities high educated community (where average schooling of adults is 7 & above years) face 20% lower odds of dying prematurely than men in the low educated communities (with where average attainment is less than three years). Similarly, those are living in the high wealth communitas (with 8 and above average wealth index) associated with 12% lower risk of death, indicate that community level wealth has a smaller impact than increasing community-level education. The description of Model 1, Model 2 and Model 3 provided in the main text of the paper.

Table S5 presents the corresponding multilevel results obtained for women. The bivariate relation between women's socio-demographic characteristics and mid-age adult mortality is similar to that of men's. Compared to uneducated women, the risk of women's mid-age mortality declines by 41% for those with at least a primary education, 61% for lower secondary, 69% for upper secondary, and 67% for post-secondary & above educated. Similarly, the risk of women's mid-age death declines significantly with higher wealth status. Both relationships are also significant at the community-level. Compared to women living in a low educated communities (with on average <3 years of schooling), women in lower-middle educated communities (with 3-< 5 average years of schooling face) have 20% lower risk, 32% lower in upper-middle (with 5-<7 years), and 47% lower risk for women in high educated communities (7 & above years of schooling). Increases in the mean average wealth index score of the community of residence were also associated with a decline in mid-age mortality. Compared to women residing in low wealth communities (with a wealth score of less than 4), those living in a lower-middle community (with an average wealth score ranging from 4 to less than 6) experience 22% lower odds of death. For women in upper middle wealth communities (with a wealth score of 6 to less than 8), the odds reduce by 32%. Women in high wealth communities (with a wealth score of 8 and above) have a 36% lower risk of dying. The description of Model 1, Model 2 and Model 3 provided in the main text of the paper.

Effects of other characteristics

Model 3 of table S4 and table S5 mortality risk by demographic, socioeconomic, health and regional characteristics controlling for other variables for males and females. The risk of mortality during mid-ages is substantially elevated among individuals with major morbidities. Specifically, individuals had asthma (4.53 times), diabetes (3.61 times), heart disease (3.52 times), and tuberculosis (2.97 times) exhibit a significantly higher risk of death compared to those without any major health conditions for males. Among females, diabetes (3.80 times), tuberculosis (3.95 times), heart disease (3.00 times), and asthma (2.80 times) are similarly associated with markedly higher mortality risks.

Marital status also plays a critical role in survival status. Among males, those who are unmarried, widowed, or separated have approximately twice the risk of death compared to their married counterparts. Among females, the mortality risk is 2.5 times higher for the unmarried, 1.8 times higher for the widowed, and 1.4 times higher for the separated, relative to currently married women.

Caste affiliation significantly influences survival prospects. Individuals from OBC (Other Backward Classes) and General caste categories show better survival outcomes than those from Scheduled Castes (SCs) and Scheduled Tribes (STs), for both males and females. Religious affiliation is significantly associated with mortality among males. Compared to Hindus, Muslim males exhibit lower mortality risk, whereas individuals from other religions show higher mortality. In contrast, religious affiliation does not significantly affect mortality outcomes among females.

Working status and type of occupation also important determinants of mid-age mortality. Economically active individuals have significantly lower mortality risk than those who are not working. Notably, individuals employed in professional, administrative, managerial, and agriculture-related occupations exhibit reduced mortality risk compared to those in production or unclassified jobs, for both sexes. The consumption of alcohol or tobacco is associated with a heightened risk of mortality. Males who consume these substances face a 34% higher risk of death, while the increase is 50% among females, compared to non-consumers. Regional factors, both in terms of rural-urban residence and broader geographic zones across India—do not exhibit a statistically significant association with midlife mortality.

Fig. S1.

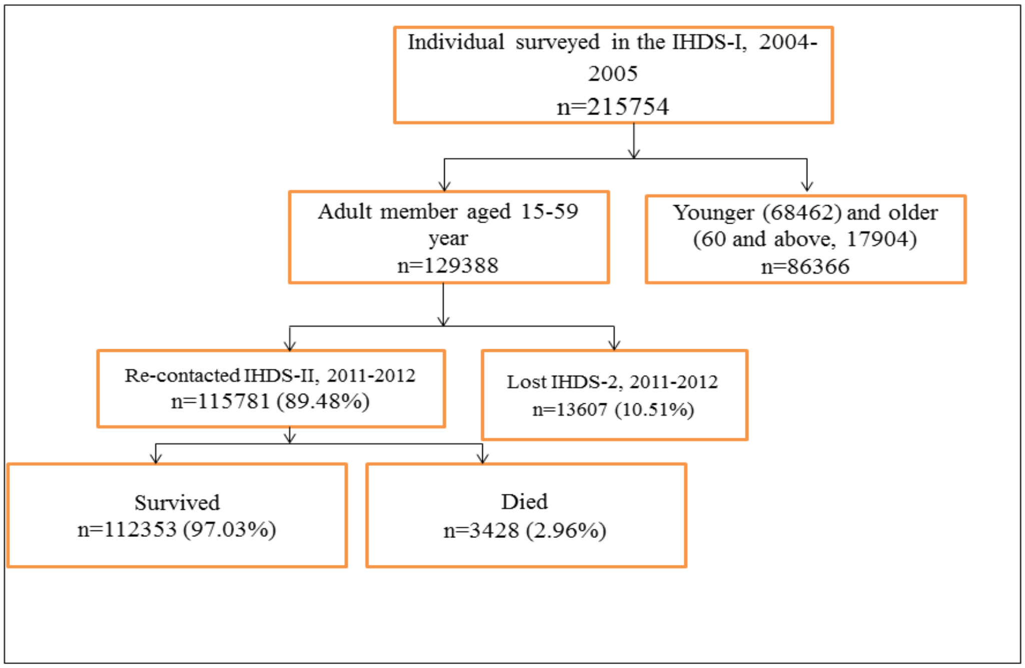

Description of the IHDS sample used for the analysis.

Fig. S2.

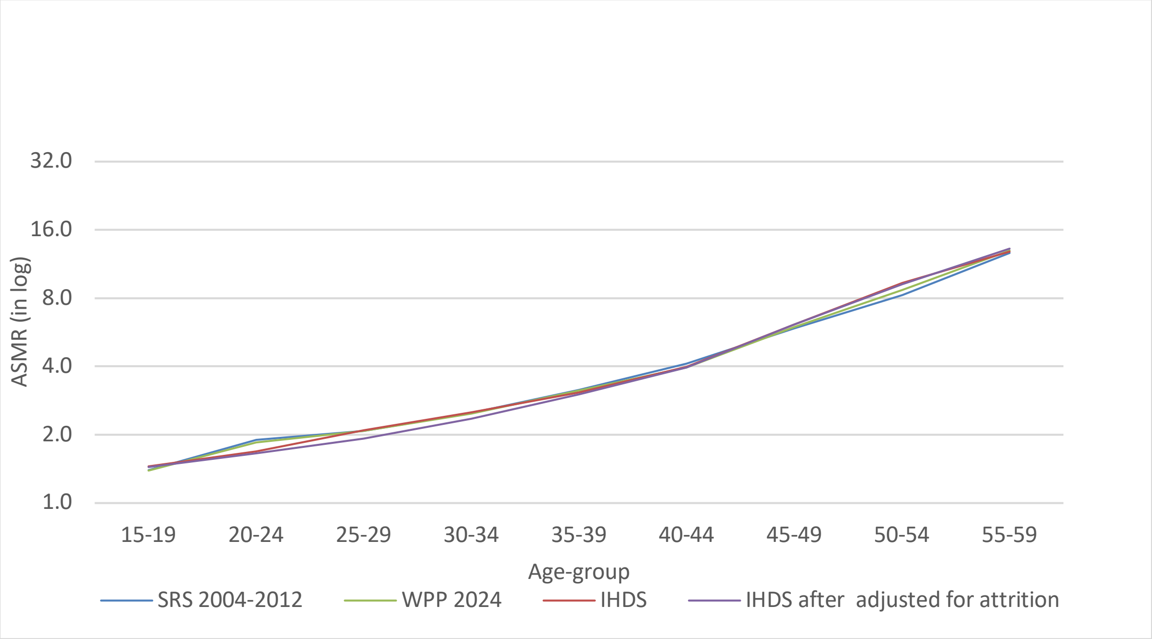

Comparison ASMR estimated from IHDS (between 2004-05 and 2011-12) with SRS estimates and United Nations 2004-2012 (12)

Fig. S3

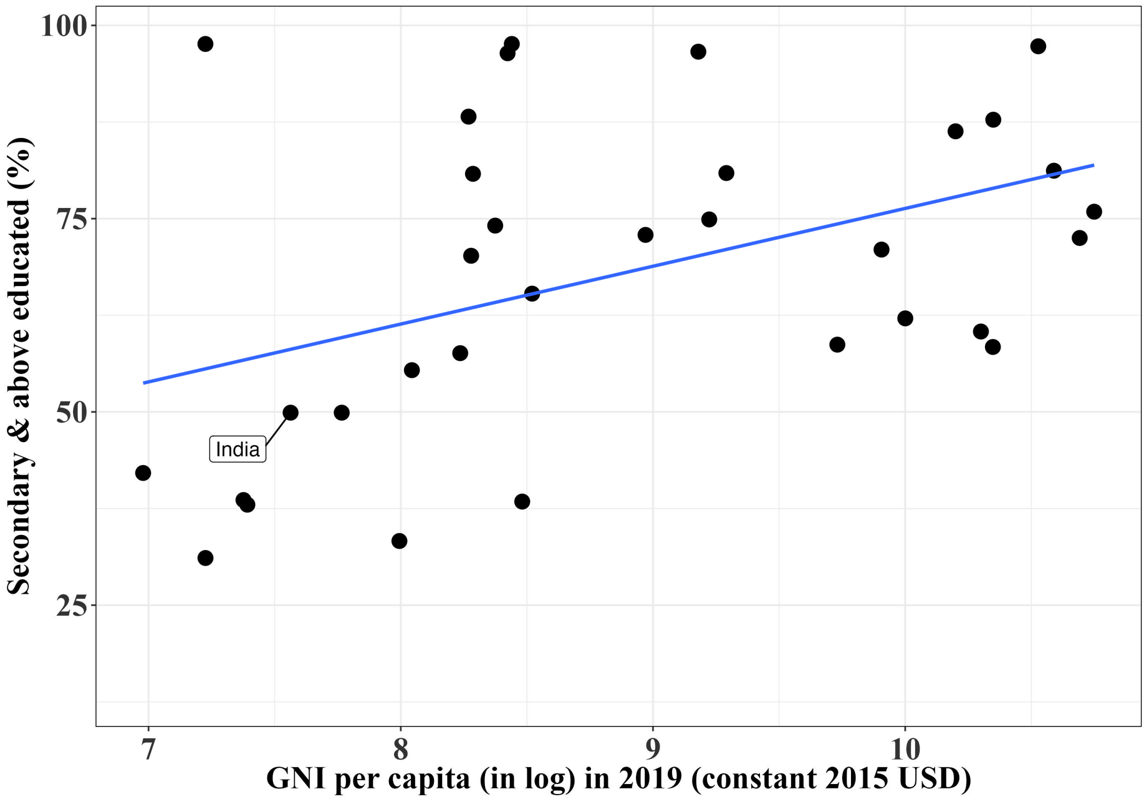

Relationship between real GDP per person (constant USD) and adults completed secondary & above educational attainment (%) in the Asian region.

Fig. S4.

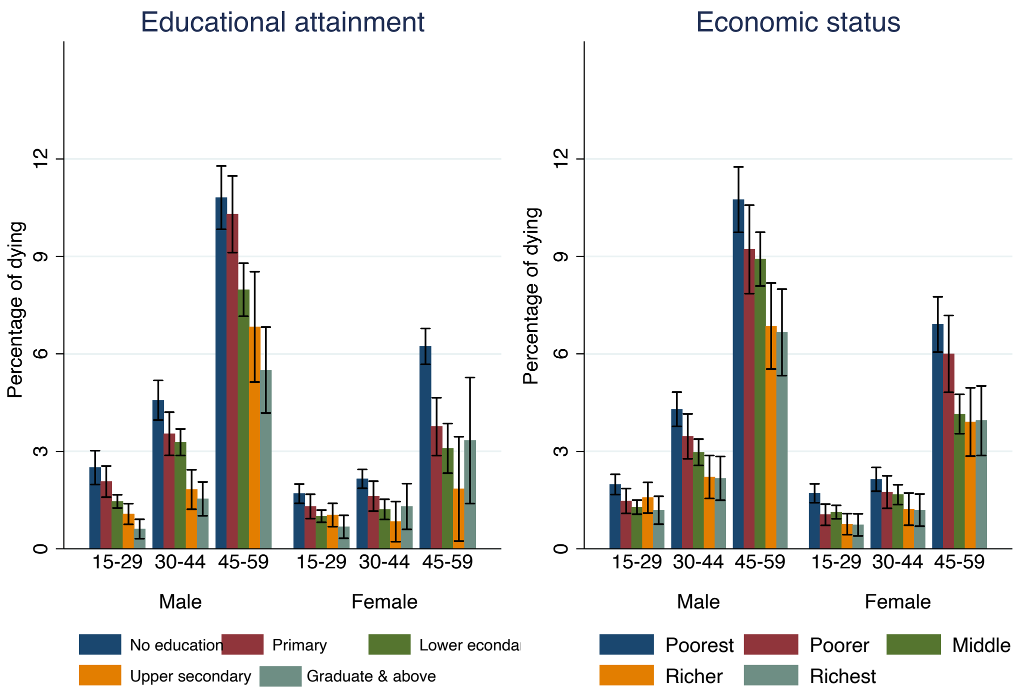

Percentage of men and women dying in mid-age between 2004-2005 and 2011-2012 by age-sex, educational attainment, and economic status (in 2004-2005).

Fig. S5.

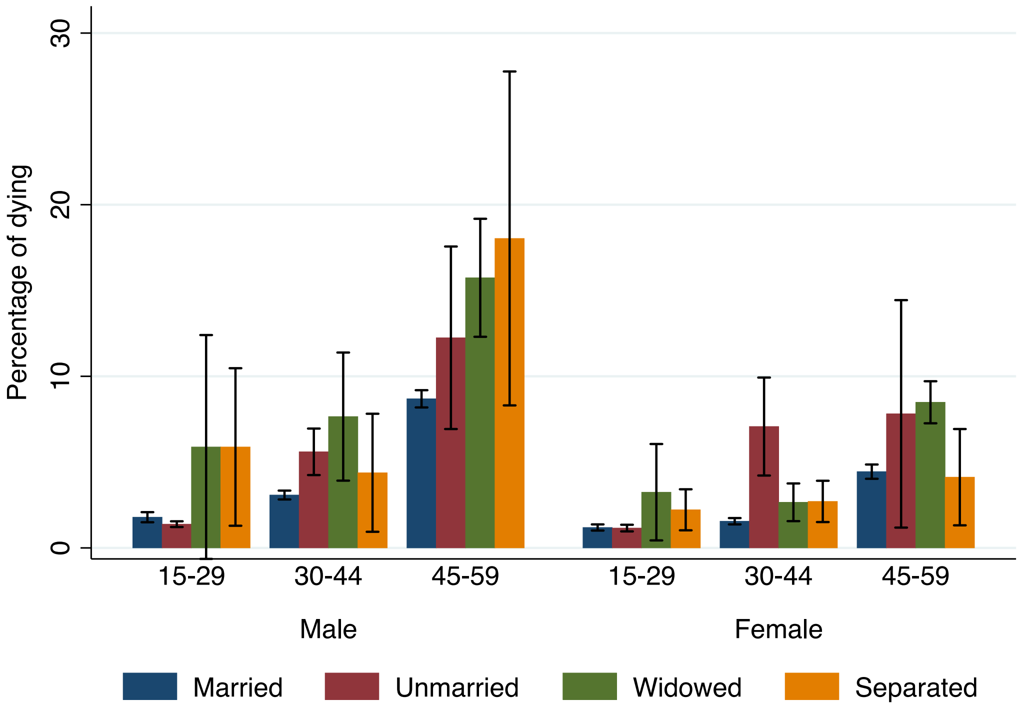

Percentage of men and women dying in mid-age between 2004-2005 and 2011-2012 by age, sex and marital status (in 2004-2005).

Fig. S6

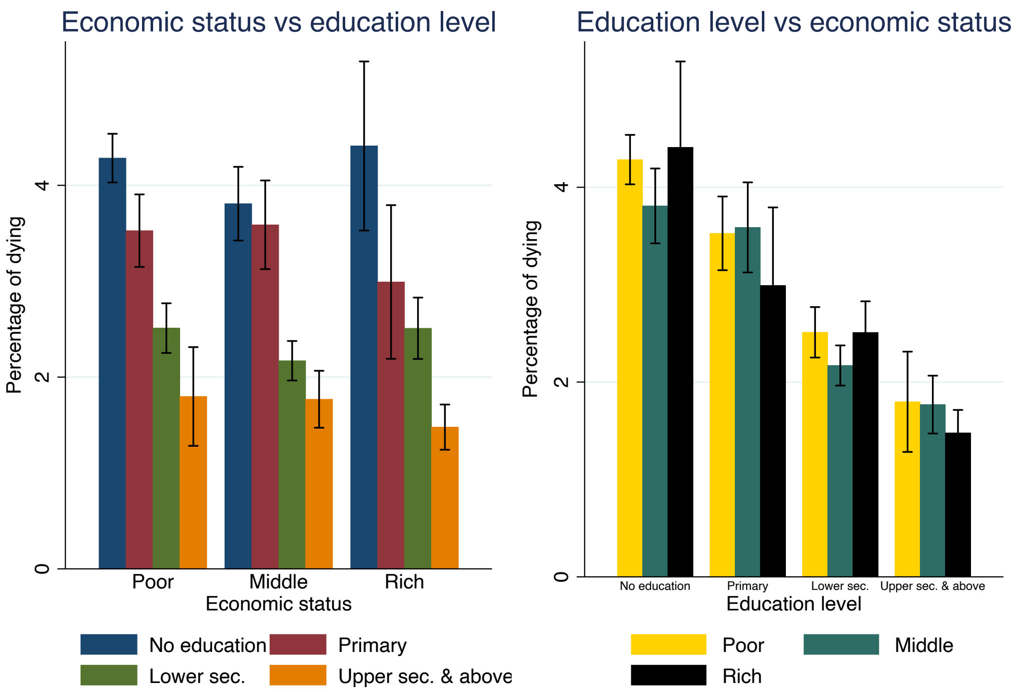

Percentage of mid-age deaths between 2004-2005 and 2011-2012 by educational attainment across economic group and vice versa

Fig. S7

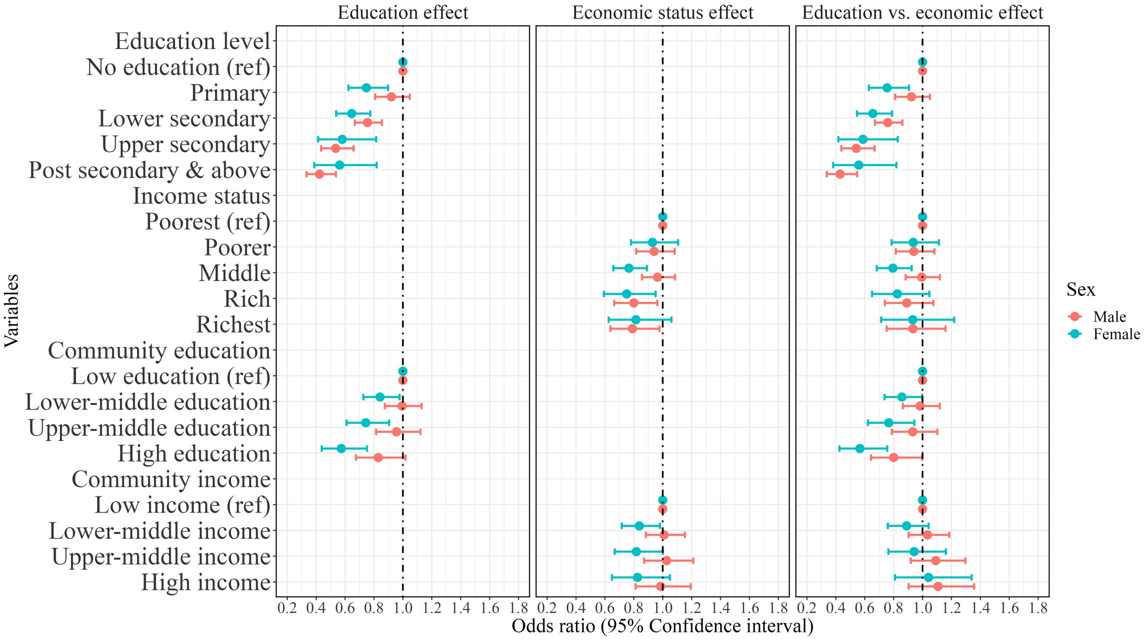

Mid-age mortality between 2004-05 and 2011-12 by education and wealth status for male-female in India (in 2004-2005), with household income as the economic indicator.

Note: Odds ratios (95% confidence intervals) are adjusted for age, health status, marital status, social group, health behaviors (consuming Alcohol/Tobacco), working status and type of occupation place of residence and region.

Fig. S8

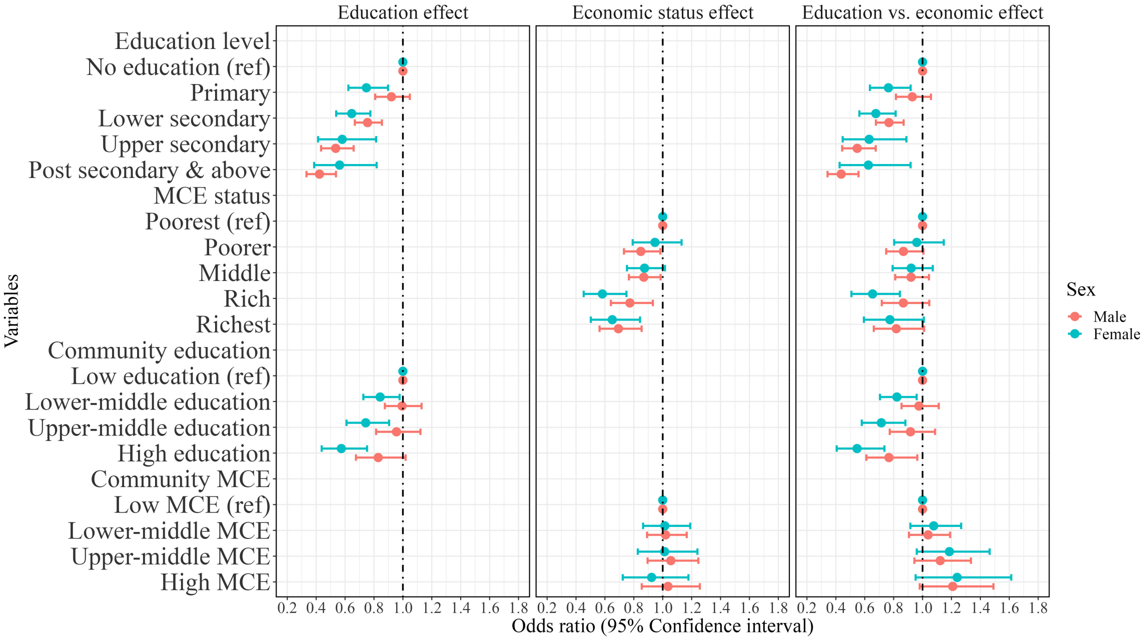

Mid-age mortality between 2004-05 and 2011-12 by education and wealth status for male-female in India (in 2004-2005), with monthly consumption expenditure as the economic indicator.

Note: Odds ratios (95% confidence intervals) are adjusted for age, health status, marital status, social group, health behaviors (consuming Alcohol/Tobacco), working status and type of occupation place of residence and region.

Table S1. Attrition in India Human Development Survey in 2011-12 for adults aged 15-59.

Variables	Re-identified (n=115,781)	Attrition (n=13,607)
**Individual variable**		
15-29	89.44	10.56
30-44	89.39	10.61
45-59	89.71	10.29
**Sex**		
Men	89.50	10.50
Women	89.47	10.53
**Morbidity**		
No	89.55	10.45
Yes	86.96	13.04
**Marital status**		
Marriage	89.82	10.18
Unmarried	88.51	11.49
widowed	89.75	10.25
Separated	92.42	7.58
**Education level**		
No education	92.64	7.36
Primary	90.45	9.55
Lower secondary	88.89	11.11
Upper secondary	86.87	13.13
Post secondary & above	82.42	17.58
**Social group**		
General	87.64	12.36
OBC	90.46	9.54
SC/ST	91.12	8.88
Muslim	87.04	12.96
Others	87.64	12.36
**Household wealth quintile**		
Poorest	94.84	5.16
Poorer	90.74	9.26
Middle	90.30	9.70
Rich	87.09	12.91
Richest	83.85	16.15
**Community level variables**		
**Mean years of community schooling**		
Low	94.13	5.87
Lower-middle	92.74	7.26
Upper-middle	89.78	10.22
High	81.30	18.70
**Place of residence**		
Rural	93.86	6.14
Urban	81.64	18.36
**Ownership of household**		
Own household	91.42	8.58
Rental house	69.60	30.40
**Work status**		
No	86.61	13.39
Yes	91.28	8.72
Total	89.49	10.51

Table S2. Percentage distribution of the sample by background characteristics in 2004-05.

Variables	Percentage	Sample size
**Morbidity**		
No Morbidity	92.97	1,07,645
Cataract	0.31	362
Tuberculosis	0.41	476
High BP	0.93	1,079
Heart disease	0.48	560
Diabetes	0.66	761
Asthma	0.59	680
Others	3.64	4,217
**Age group**		
15-29	45.92	53,169
30-44	32.81	37,983
45-59	21.27	24,629
**Sex**		
Men	50.08	57,988
Women	49.92	57,793
**Marital status**		
Marriage	68.02	78,757
Unmarried	26.97	31,224
widowed	3.22	3,733
Separated	1.79	2,067
**Education level**		
No education	33.90	39,254
Primary	15.43	17,865
Lower secondary	34.85	40,345
Upper secondary	8.68	10,047
Post-secondary & above	7.14	8,270
**Wealth group**		
Poorest	33.90	39,250
Poorer	15.43	17,866
Middle	34.84	40,343
Rich	8.68	10,049
Richest	7.15	8,274
**Income group**		
Poorest	33.90	39,249
Poorer	15.43	17,867
Middle	34.84	40,343
Rich	8.68	10,048
Richest	7.15	8,274
**Monthly consumption expenditure (MCE) group**		
Poorest	33.89	39,238
Poorer	15.44	17,878
Middle	34.84	40,344
Rich	8.68	10,047
Richest	7.15	8,274
**Mean year of community schooling**		
Low	26.63	30,832
Lower-middle	41.28	47,794
Upper-middle	19.70	22,804
High	12.40	14,352
**Average community wealth quintile**		
Low	28.65	33,169
Lower-middle	33.58	38,875
Upper-middle	20.65	23,912
High	17.12	19,825
**Average community income**		
Low	26.63	30,828
Lower-middle	41.28	47,797
Upper-middle	19.69	22,793

High	12.41	14,363
**Average community MCE**		
Low		
Lower-middle	26.63	30,832
Upper-middle	41.28	47,792
High	19.69	22,794
**Social group**	12.41	14,363
SC/ST		
OBC	41.41	47,941
Others	29.28	33,896
**Religion**		
Hindu	82.25	95,229
Muslim	11.92	13,806
Others	5.83	6,746
**Health Behaviour (consuming Alcohol/Tobacco)**		
No	77.59	89,829
Yes	22.41	25,952
**Type of occupation**		
Not Working	35.78	41,432
Professional & Admin/Manager	2.26	2,611
Clerical/Sales/Service	4.73	5,472
Farmers	15.75	18,233
Production	13.64	15,796
OCC Not Classified	27.77	32,151
Other	0.07	86
**Region**		
North	14.49	16,776
Central	22.84	26,447
East-Northeast	25.05	29,004
West	16.08	18,614
South	21.54	24,940
**Place of residence**		
Rural	73.31	84,882
Urban	26.69	30,899
Total		1,15,781

Table S3. Percentage of mid-age adults (15-59 years) in 2004-05 dying before 2011-12 by demographic and socioeconomic characteristics.

Variables	Died		Survived		Total
	Percentage of died	number	%	number	
**Morbidity**					
No Morbidity	2.6 (2.5-2.69)	2797 (2691-2896)	97.4 (97.31-97.5)	104848 (104749-104954)	107645 (107440-107850)
Cataract	9.29 (6.11-12.48)	34 (22-45)	90.71 (87.52-93.89)	328 (317-340)	362 (339-385)
Tuberculosis	10.48 (7.42-13.54)	50 (35-64)	89.52 (86.46-92.58)	426 (412-441)	476 (447-505)
High BP	6.46 (5.03-7.89)	70 (54-85)	93.54 (92.11-94.97)	1010 (994-1025)	1079 (1048-1110)
Heart disease	8.56 (6.14-10.97)	48 (34-61)	91.44 (89.03-93.86)	512 (499-526)	560 (533-587)
Diabetes	12.25 (9.9-14.6)	93 (75-111)	87.75 (85.4-90.1)	668 (650-686)	761 (725-797)
Asthma	14.86 (11.77-17.94)	101 (80-122)	85.14 (82.06-88.23)	579 (558-600)	680 (638-722)
Others	8.15 (7.27-9.03)	344 (307-381)	91.85 (90.97-92.73)	3873 (3836-3910)	4217 (4143-4291)
**Age group**					
15-29	1.42 (1.32-1.52)	753 (702-808)	98.58 (98.48-98.68)	52417 (52361-52467)	53169 (53063-53275)
30-44	2.52 (2.36-2.67)	955 (896-1014)	97.48 (97.33-97.64)	37027 (36969-37087)	37983 (37865-38101)
45-59	7.42 (7.09-7.75)	1828 (1746-1909)	92.58 (92.25-92.91)	22801 (22720-22883)	24629 (24466-24792)
**Sex**					
Men	3.72 (3.57-3.88)	2159 (2070-2250)	96.28 (96.12-96.43)	55829 (55738-55918)	57988 (57808-58168)
Women	2.38 (2.26-2.51)	1377 (1306-1451)	97.62 (97.49-97.74)	56416 (56342-56487)	57793 (57649-57938)
**Marital status**					
Marriage	3.38 (3.25-3.5)	2660 (2560-2756)	96.62 (96.5-96.75)	76097 (76001-76197)	78757 (78560-78953)
Unmarried	1.54 (1.4-1.67)	480 (437-521)	98.46 (98.33-98.6)	30744 (30703-30787)	31224 (31140-31308)
widowed	8.33 (7.44-9.23)	311 (278-345)	91.67 (90.77-92.56)	3422 (3388-3455)	3733 (3666-3800)
Separated	4.11 (3.19-5.04)	85 (66-104)	95.89 (94.96-96.81)	1982 (1963-2001)	2067 (2029-2105)
**Education level**					
No education	4.37 (4.16-4.58)	1717 (1633-1798)	95.63 (95.42-95.84)	37538 (37456-37621)	39254 (39089-39419)
Primary	3.42 (3.15-3.69)	611 (563-659)	96.58 (96.31-96.85)	17253 (17206-17302)	17865 (17769-17961)
Lower secondary	2.28 (2.14-2.42)	919 (863-976)	97.72 (97.58-97.86)	39425 (39369-39482)	40345 (40232-40458)
Upper secondary	1.5 (1.28-1.73)	151 (129-174)	98.5 (98.27-98.72)	9896 (9873-9918)	10047 (10002-10092)
Post-secondary & above	1.66 (1.4-1.92)	137 (116-159)	98.34 (98.08-98.6)	8133 (8111-8154)	8270 (8227-8313)
**Wealth group**					
Poorest	3.86 (3.66-4.07)	1516 (1437-1597)	96.14 (95.93-96.34)	37734 (37653-37813)	39250 (39090-39410)
Poorer	3 (2.74-3.27)	536 (490-584)	97 (96.73-97.26)	17329 (17282-17376)	17866 (17772-17960)
Middle	2.7 (2.54-2.85)	1087 (1025-1150)	97.3 (97.15-97.46)	39256 (39193-39318)	40343 (40218-40468)
Rich	2.24 (1.97-2.51)	225 (198-252)	97.76 (97.49-98.03)	9824 (9797-9851)	10049 (9995-10103)
Richest	2.07 (1.8-2.33)	171 (149-193)	97.93 (97.67-98.2)	8103 (8081-8125)	8274 (8230-8318)
**Income group**					
Poorest	3.6 (3.41-3.8)	1414 (1337-1491)	96.4 (96.2-96.59)	37835 (37758-37912)	39249 (39095-39403)
Poorer	3.23 (2.96-3.5)	577 (529-625)	96.77 (96.5-97.04)	17290 (17242-17338)	17867 (17771-17963)
Middle	2.77 (2.62-2.93)	1119 (1056-1182)	97.23 (97.07-97.38)	39224 (39161-39288)	40343 (40217-40470)

Rich	2.34 (2.07-2.61)	235 (208-262)	97.66 (97.39-97.93)	9813 (9786-9840)	10048 (9994-10102)
Richest	2.32 (2.02-2.61)	192 (167-216)	97.68 (97.39-97.98)	8083 (8058-8107)	8274 (8225-8323)
**Monthly consumption expenditure (MCE) group**					
Poorest	3.33 (3.14-3.51)	1305 (1232-1378)	96.67 (96.49-96.86)	37933 (37860-38006)	39238 (39092-39384)
Poorer	2.92 (2.67-3.18)	522 (476-568)	97.08 (96.82-97.33)	17356 (17310-17402)	17878 (17786-17970)
Middle	3.04 (2.88-3.21)	1228 (1161-1295)	96.96 (96.79-97.12)	39116 (39049-39183)	40344 (40210-40478)
Rich	2.53 (2.25-2.82)	255 (226-283)	97.47 (97.18-97.75)	9792 (9764-9821)	10047 (9990-10104)
Richest	2.73 (2.42-3.05)	226 (200-252)	97.27 (96.95-97.58)	8048 (8021-8074)	8274 (8221-8326)
**Mean year of community schooling**					
Low	3.48 (3.26-3.7)	1073 (1005-1141)	96.52 (96.3-96.74)	29758 (29691-29827)	30832 (30696-30968)
Lower-middle	3.19 (3.02-3.35)	1522 (1443-1601)	96.81 (96.65-96.98)	46272 (46193-46351)	47794 (47636-47952)
Upper-middle	2.69 (2.49-2.88)	613 (568-657)	97.31 (97.12-97.51)	22191 (22147-22236)	22804 (22715-22893)
High	2.28 (2.06-2.5)	327 (296-359)	97.72 (97.5-97.94)	14024 (13993-14056)	14352 (14289-14415)
**Average community wealth quintile**					
Low	3.45 (3.23-3.66)	1144 (1071-1214)	96.55 (96.34-96.77)	32025 (31955-32098)	33169 (33026-33312)
Lower-middle	3.1 (2.91-3.28)	1204 (1131-1275)	96.9 (96.72-97.09)	37672 (37600-37744)	38875 (38731-39019)
Upper-middle	2.87 (2.67-3.06)	686 (638-732)	97.13 (96.94-97.33)	23227 (23180-23274)	23912 (23818-24006)
High	2.54 (2.35-2.72)	503 (466-539)	97.46 (97.28-97.65)	19322 (19286-19359)	19825 (19752-19898)
**Average community income**					
Low	3.44 (3.21-3.68)	1062 (989-1134)	96.56 (96.32-96.79)	29766 (29693-29839)	30828 (30682-30973)
Lower-middle	3.05 (2.9-3.21)	1458 (1384-1533)	96.95 (96.79-97.1)	46339 (46264-46413)	47797 (47648-47946)
Upper-middle	2.87 (2.67-3.07)	655 (609-700)	97.13 (96.93-97.33)	22138 (22093-22184)	22793 (22702-22884)
High	2.52 (2.29-2.74)	361 (329-394)	97.48 (97.26-97.71)	14002 (13969-14034)	14363 (14298-14428)
**Average community MCE**					
Low					
Lower-middle	3.2 (2.99-3.42)	988 (923-1053)	96.8 (96.58-97.01)	29844 (29779-29910)	30832 (30702-30963)
Upper-middle	3.03 (2.87-3.19)	1447 (1370-1523)	96.97 (96.81-97.13)	46345 (46268-46422)	47792 (47638-47945)
High	3.19 (2.97-3.4)	727 (678-776)	96.81 (96.6-97.03)	22067 (22018-22116)	22794 (22696-22892)
**Social group**	2.61 (2.39-2.83)	374 (343-406)	97.39 (97.17-97.61)	13989 (13957-14020)	14363 (14300-14426)
SC/ST					
OBC	2.98 (2.82-3.14)	1429 (1352-1505)	97.02 (96.86-97.18)	46512 (46436-46589)	47941 (47788-48094)
Others	2.45 (2.29-2.61)	830 (776-885)	97.55 (97.39-97.71)	33066 (33011-33120)	33896 (33787-34005)
**Religion**					
Hindu	3.12 (3-3.23)	2967 (2857-3076)	96.88 (96.77-97)	92262 (92153-92372)	95229 (95010-95448)
Muslim	2.46 (2.2-2.72)	339 (304-376)	97.54 (97.28-97.8)	13467 (13430-13502)	13806 (13734-13878)
Others	3.41 (3.02-3.79)	230 (204-256)	96.59 (96.21-96.98)	6516 (6490-6542)	6746 (6694-6798)

**Health Behaviour (consuming Alcohol/Tobacco)**					
No	2.31 (2.22-2.41)	2078 (1994-2165)	97.69 (97.59-97.78)	87750 (87664-87835)	89829 (89658-90000)
Yes	5.62 (5.33-5.9)	1458 (1383-1531)	94.38 (94.1-94.67)	24495 (24421-24569)	25952 (25804-26100)
**Type of occupation**					
Not Working	2.6 (2.45-2.75)	1077 (1015-1139)	97.4 (97.25-97.55)	40356 (40293-40417)	41432 (41308-41556)
Professional & Admin/Manager	2.65 (2.08-3.23)	69 (54-84)	97.35 (96.77-97.92)	2541 (2527-2557)	2611 (2581-2641)
Clerical/Sales/Service	3.91 (3.42-4.4)	214 (187-241)	96.09 (95.6-96.58)	5258 (5231-5285)	5472 (5418-5526)
Farmers	3.62 (3.34-3.9)	660 (609-711)	96.38 (96.1-96.66)	17573 (17522-17624)	18233 (18131-18335)
Production	3.6 (3.3-3.89)	568 (521-614)	96.4 (96.11-96.7)	15228 (15182-15275)	15796 (15703-15889)
OCC Not Classified	2.93 (2.75-3.12)	942 (884-1003)	97.07 (96.88-97.25)	31209 (31148-31267)	32151 (32032-32270)
Other	6.27 (0.42-12.12)	5 (0-10)	93.73 (87.88-99.58)	81 (76-86)	86 (76-96)
**Region**					
North	2.98 (2.78-3.18)	499 (466-533)	97.02 (96.82-97.22)	16277 (16243-16310)	16776 (16709-16843)
Central	3.15 (2.93-3.37)	833 (775-891)	96.85 (96.63-97.07)	25614 (25556-25672)	26447 (26331-26563)
East-Northeast	2.98 (2.77-3.2)	866 (803-928)	97.02 (96.8-97.23)	28138 (28076-28201)	29004 (28879-29129)
West	2.73 (2.48-2.98)	508 (462-555)	97.27 (97.02-97.52)	18106 (18059-18152)	18614 (18521-18707)
South	3.33 (3.11-3.55)	830 (776-885)	96.67 (96.45-96.89)	24110 (24055-24164)	24940 (24831-25049)
**Place of residence**					
Rural	3.18 (3.05-3.3)	2695 (2589-2801)	96.82 (96.7-96.95)	82187 (82081-82293)	84882 (84670-85094)
Urban	2.72 (2.56-2.88)	841 (791-890)	97.28 (97.12-97.44)	30058 (30009-30108)	30899 (30800-30998)
**Total**	3.05 (2.95-3.15)	3536 (3416-3647)	96.95 (96.85-97.05)	112245 (112134-112365)	115781 (115550-116012)

Note: Confidence interval in parenthesis

Table S4. Multilevel model results: Odds ratios for male mid-age death between 2004-05 and 2011-12.

Variables	Bivariate	Model 1 (Education effect)	Model 2 (Economic status effect)	Model 3 (Education vs Economic status effect)
**Morbidity**				
No Morbidity				
Cataract	5.212***(3.172-8.564)	2.138***(1.277-3.581)	2.115***(1.262-3.545)	2.07***(1.235-3.471)
Tuberculosis	5.06***(3.385-7.566)	3.035***(2.001-4.602)	3.124***(2.059-4.741)	2.97***(1.958-4.505)
High BP	2.964***(2.047-4.291)	1.728***(1.183-2.524)	1.735***(1.188-2.533)	1.782***(1.22-2.605)
Heart disease	5.433***(3.675-8.03)	3.457***(2.313-5.165)	3.456***(2.314-5.161)	3.52***(2.356-5.259)
Diabetes	6.071***(4.61-7.997)	3.486***(2.615-4.647)	3.534***(2.65-4.713)	3.607***(2.703-4.814)
Asthma	9.519***(6.98-12.982)	4.606***(3.331-6.37)	4.622***(3.341-6.395)	4.525***(3.27-6.262)
Others	3.999***(3.381-4.731)	2.74***(2.302-3.262)	2.773***(2.329-3.301)	2.707***(2.274-3.223)
**Age squire (in log scale)**	3.091***(2.884-3.313)	3.254***(2.952-3.585)	3.446***(3.131-3.791)	3.328***(3.018-3.669)
Marital status				
Marriage				
Unmarried	0.353***(0.314-0.398)	1.815***(1.52-2.166)	1.768***(1.483-2.109)	1.82***(1.524-2.173)
widowed	2.993***(2.368-3.783)	1.664***(1.3-2.13)	1.705***(1.332-2.184)	1.627***(1.27-2.084)
Separated	1.69**(1.097-2.603)	2.001***(1.276-3.136)	1.968***(1.254-3.09)	1.957***(1.247-3.071)
Education level				
**No education**				
Primary	0.798***(0.705-0.903)	0.92 (0.808-1.048)		0.95 (0.834-1.083)
Lower secondary	0.487***(0.438-0.542)	0.755***(0.667-0.855)		0.809***(0.713-0.918)
Upper secondary	0.31***(0.257-0.375)	0.535***(0.434-0.659)		0.594***(0.48-0.735)
Post-secondary & above	0.292***(0.238-0.359)	0.423***(0.333-0.537)		0.479***(0.374-0.613)
**Wealth group**				
Poorest				
Poorer	0.8***(0.698-0.916)		0.798***(0.69-0.923)	0.819***(0.708-0.948)
Middle	0.719***(0.647-0.798)		0.673***(0.588-0.769)	0.725***(0.632-0.831)
Rich	0.626***(0.529-0.74)		0.539***(0.438-0.662)	0.626***(0.507-0.773)
Richest	0.581***(0.486-0.694)		0.488***(0.389-0.614)	0.622***(0.491-0.788)
**Mean years of community schooling**				
Low				
Lower-middle	0.953 (0.847-1.073)	0.994 (0.875-1.129)		0.971 (0.848-1.112)
Upper-middle	0.912 (0.798-1.042)	0.956 (0.815-1.121)		0.91 (0.759-1.092)
High	0.799***(0.685-0.933)	0.829*(0.675-1.019)		0.767**(0.604-0.975)
**Average community wealth index**				
Low				
Lower-middle	0.956 (0.843-1.084)		1.1 (0.96-1.261)	1.126 (0.976-1.298)
Upper-middle	0.926 (0.814-1.055)		1.178*(0.998-1.39)	1.251**(1.043-1.501)

High	0.876*(0.767-1.001)		1.181 (0.964-1.448)	1.376***(1.087-1.743)
**Social group**				
SC/ST				
OBC	0.767***(0.692-0.849)	0.832***(0.744-0.93)	0.83***(0.742-0.928)	0.847***(0.757-0.948)
Others	0.638***(0.57-0.714)	0.722***(0.635-0.821)	0.696***(0.612-0.792)	0.748***(0.657-0.852)
**Religion**				
Hindu				
Muslim	0.727***(0.623-0.848)	0.785***(0.664-0.929)	0.855*(0.724-1.009)	0.782***(0.661-0.925)
Others	1.322***(1.136-1.539)	1.331***(1.132-1.564)	1.374***(1.165-1.62)	1.344***(1.139-1.584)
**Health Behavior (consuming Alcohol/Tobacco)**				
No				
Yes	2.382***(2.183-2.601)	1.37***(1.238-1.516)	1.402***(1.267-1.552)	1.342***(1.212-1.486)
**Type of occupation**				
Not Working				
Professional & Admin/Manager	0.906 (0.693-1.183)	0.566***(0.423-0.758)	0.442***(0.333-0.585)	0.577***(0.43-0.773)
Clerical/Sales/Service	1.517***(1.284-1.793)	0.797**(0.664-0.957)	0.75***(0.625-0.9)	0.8**(0.666-0.961)
Farmers	1.356***(1.176-1.563)	0.554***(0.47-0.654)	0.556***(0.47-0.657)	0.527***(0.446-0.623)
Production	1.253***(1.096-1.433)	0.614***(0.528-0.714)	0.617***(0.531-0.718)	0.593***(0.509-0.69)
OCC Not Classified	1.11 (0.973-1.265)	0.573***(0.494-0.664)	0.589***(0.508-0.682)	0.582***(0.502-0.674)
**Region**				
North				
Central	0.984 (0.857-1.13)	0.957 (0.826-1.109)	0.889 (0.762-1.037)	0.949 (0.812-1.109)
East-Northeast	0.965 (0.84-1.109)	0.822***(0.712-0.95)	0.74***(0.633-0.865)	0.828**(0.704-0.973)
West	0.953 (0.816-1.113)	0.951 (0.809-1.117)	0.891 (0.756-1.05)	0.958 (0.811-1.132)
South	1.097 (0.961-1.252)	0.973 (0.844-1.121)	0.902 (0.776-1.048)	0.994 (0.849-1.165)
**Place of residence**				
Rural				
Urban	0.905**(0.821-0.998)	1.083 (0.959-1.222)	1.036 (0.911-1.179)	1.085 (0.953-1.236)
Random effects				
Level 2 (Community)		0.097(0.046-0.205)	0.11(0.056-0.213)	0.01(0.048-0.207)

Note: *p < = .05, **p < = .01, ***p < = .001, Confidence interval in parenthesis

Table S5. Multilevel model results: Odds ratios of female mid-age death between 2004-05 and 2011-12.

Variables	Bivariate	Model 1 (Education effect)	Model 2 (Economic status effect)	Model 3 (Education vs Economic status effect)
**Morbidity**				
No Morbidity				
Cataract	4.469***(2.618-7.629)	2.085***(1.207-3.601)	2.126***(1.23-3.673)	2.072***(1.199-3.582)
Tuberculosis	5.789***(3.529-9.497)	4***(2.415-6.624)	4.146***(2.501-6.874)	3.954***(2.387-6.55)
High BP	1.771***(1.181-2.656)	1.148 (0.76-1.733)	1.137 (0.753-1.717)	1.162 (0.77-1.756)
Heart disease	4.472***(2.925-6.837)	3.015***(1.954-4.651)	2.949***(1.913-4.546)	3.017***(1.955-4.654)
Diabetes	5.379***(3.706-7.808)	3.673***(2.498-5.4)	3.551***(2.416-5.219)	3.788***(2.574-5.575)
Asthma	4.91***(3.177-7.589)	2.858***(1.828-4.466)	2.877***(1.84-4.497)	2.827***(1.809-4.418)
Others	3.064***(2.521-3.724)	2.361***(1.935-2.88)	2.363***(1.937-2.883)	2.357***(1.932-2.876)
**Age** squire (in log scale)	2.36***(2.171-2.566)	2.388***(2.131-2.676)	2.559***(2.291-2.858)	2.433***(2.17-2.729)
**Marital status**				
Marriage				
Unmarried	0.635***(0.536-0.752)	2.527***(2.01-3.176)	2.269***(1.812-2.841)	2.534***(2.016-3.187)
widowed	3.303***(2.813-3.879)	1.818***(1.53-2.16)	1.858***(1.564-2.208)	1.801***(1.515-2.14)
Separated	1.295 (0.938-1.787)	1.493**(1.077-2.07)	1.46**(1.052-2.025)	1.494**(1.077-2.073)
**Education level**				
No education				
Primary	0.588***(0.495-0.699)	0.748***(0.623-0.897)		0.774***(0.644-0.93)
Lower secondary	0.389***(0.335-0.452)	0.646***(0.538-0.775)		0.685***(0.568-0.826)
Upper secondary	0.314***(0.231-0.427)	0.58***(0.413-0.815)		0.627***(0.443-0.886)
Post-secondary & above	0.335***(0.241-0.465)	0.562***(0.386-0.818)		0.607**(0.412-0.895)
**Wealth group**				
Poorest				
Poorer	0.772***(0.649-0.919)		0.849*(0.706-1.02)	0.867 (0.721-1.043)
Middle	0.65***(0.568-0.743)		0.727***(0.615-0.86)	0.779***(0.657-0.923)
Rich	0.543***(0.435-0.678)		0.583***(0.446-0.761)	0.672***(0.512-0.882)
Richest	0.551***(0.438-0.692)		0.595***(0.447-0.794)	0.74**(0.549-0.997)
**Mean years of community schooling**				
Low				
Lower-middle	0.797***(0.694-0.917)	0.843**(0.726-0.977)		0.871*(0.744-1.02)
Upper-middle	0.678***(0.575-0.799)	0.743***(0.61-0.905)		0.755**(0.602-0.945)
High	0.528***(0.431-0.647)	0.574***(0.438-0.752)		0.542***(0.397-0.741)
**Average community wealth index**				
Low				
Lower-middle	0.776***(0.668-0.901)		0.861*(0.732-1.012)	0.925 (0.782-1.094)

Upper-middle	0.682***(0.581-0.8)		0.837*(0.682-1.027)	0.995 (0.796-1.243)
High	0.642***(0.544-0.757)		0.838 (0.649-1.083)	1.203 (0.895-1.616)
**Social group**				
SC/ST				
OBC	0.725***(0.636-0.828)	0.73***(0.634-0.841)	0.725***(0.629-0.836)	0.75***(0.651-0.865)
Others	0.646***(0.56-0.746)	0.725***(0.616-0.855)	0.671***(0.57-0.791)	0.753***(0.637-0.889)
**Religion**				
Hindu				
Muslim	1.018 (0.855-1.212)	1.003 (0.829-1.214)	1.131 (0.937-1.364)	0.998 (0.824-1.208)
Others	0.985 (0.792-1.226)	0.975 (0.778-1.223)	0.951 (0.756-1.197)	0.963 (0.765-1.212)
**Health Behaviour (consuming Alcohol/Tobacco)**				
No				
Yes	2.566***(2.187-3.01)	1.542***(1.3-1.83)	1.556***(1.311-1.847)	1.514***(1.276-1.797)
**Type of occupation**				
Not Working				
Professional & Admin/Manager	0.491**(0.243-0.99)	0.541*(0.262-1.117)	0.438**(0.216-0.891)	0.542*(0.262-1.121)
Clerical/Sales/Service	1.024 (0.688-1.524)	0.622**(0.414-0.935)	0.598**(0.398-0.899)	0.605**(0.402-0.91)
Farmers	1.098 (0.925-1.304)	0.63***(0.519-0.764)	0.618***(0.508-0.753)	0.589***(0.484-0.718)
Production	1.042 (0.787-1.379)	0.646***(0.484-0.861)	0.642***(0.481-0.858)	0.619***(0.463-0.827)
OCC Not Classified	0.939 (0.821-1.074)	0.66***(0.569-0.765)	0.668***(0.576-0.776)	0.659***(0.567-0.764)
**Region**				
North				
Central	1.287***(1.084-1.528)	1.14 (0.951-1.365)	1.071 (0.888-1.294)	1.14 (0.944-1.378)
East-Northeast	1.199**(1.007-1.428)	1.004 (0.838-1.203)	0.839*(0.691-1.02)	1 (0.817-1.225)
West	0.855 (0.692-1.058)	0.897 (0.721-1.116)	0.801**(0.642-0.999)	0.919 (0.734-1.15)
South	1.145 (0.965-1.359)	1.063 (0.886-1.276)	0.87 (0.719-1.052)	1.084 (0.886-1.326)
**Place of residence**				
Rural				
Urban	0.847***(0.747-0.96)	0.981 (0.841-1.146)	0.926 (0.785-1.093)	0.999 (0.846-1.179)
Random effects				
Level 2 (Community)		0.028(.0001-1.252)	0.05(0.005-0.436)	0.03(0.001-1.031)

Note: *p < = .05, **p < = .01, ***p < = .001, Confidence interval in parenthesis

Table S6. Multilevel model results: Odds ratios for male mid-age death between 2004-05 and 2011-12, with household income as the economic indicator.

**Variables**	Bivariate	Model 1 (Education effect)	Model 2 (Economic status effect)	Model 3 (Education vs Economic status effect)
**Morbidity**				
No Morbidity				
Cataract	5.212***(3.172-8.564)	2.138***(1.277-3.581)	2.219***(1.326-3.714)	2.126***(1.269-3.564)
Tuberculosis	5.06***(3.385-7.566)	3.035***(2.001-4.602)	3.256***(2.148-4.935)	3.048***(2.01-4.622)
High BP	2.964***(2.047-4.291)	1.728***(1.183-2.524)	1.647***(1.129-2.403)	1.734***(1.187-2.534)
Heart disease	5.433***(3.675-8.03)	3.457***(2.313-5.165)	3.355***(2.246-5.01)	3.465***(2.319-5.179)
Diabetes	6.071***(4.61-7.997)	3.486***(2.615-4.647)	3.327***(2.498-4.43)	3.504***(2.628-4.672)
Asthma	9.519***(6.98-12.982)	4.606***(3.331-6.37)	4.717***(3.413-6.519)	4.6***(3.326-6.363)
Others	3.999***(3.381-4.731)	2.74***(2.302-3.262)	2.804***(2.356-3.338)	2.736***(2.298-3.257)
**Age** squire (in log scale)	3.091***(2.884-3.313)	3.254***(2.952-3.585)	3.409***(3.098-3.751)	3.264***(2.961-3.598)
**Marital status**				
Marriage				
Unmarried	0.353***(0.314-0.398)	1.815***(1.52-2.166)	1.789***(1.499-2.133)	1.822***(1.526-2.175)
widowed	2.993***(2.368-3.783)	1.664***(1.3-2.13)	1.779***(1.391-2.276)	1.659***(1.296-2.124)
Separated	1.69**(1.097-2.603)	2.001***(1.276-3.136)	2.067***(1.318-3.241)	1.999***(1.275-3.135)
**Education level**				
No education				
Primary	0.798***(0.705-0.903)	0.92 (0.808-1.048)		0.923 (0.81-1.051)
Lower secondary	0.487***(0.438-0.542)	0.755***(0.667-0.855)		0.76***(0.671-0.86)
Upper secondary	0.31***(0.257-0.375)	0.535***(0.434-0.659)		0.541***(0.439-0.668)
Post-secondary & above	0.292***(0.238-0.359)	0.423***(0.333-0.537)		0.43***(0.337-0.547)
**Income group**				
Poorest				
Poorer	0.894 (0.78-1.025)		0.94 (0.816-1.083)	0.939 (0.815-1.082)
Middle	0.867***(0.781-0.963)		0.964 (0.857-1.085)	0.994 (0.883-1.119)
Rich	0.722***(0.613-0.851)		0.8**(0.664-0.963)	0.891 (0.739-1.075)
Richest	0.686***(0.572-0.822)		0.79**(0.639-0.978)	0.933 (0.752-1.159)
**Mean years of community schooling**				
Low				
Lower-middle	0.953 (0.847-1.073)	0.994 (0.875-1.129)		0.983 (0.863-1.119)
Upper-middle	0.912 (0.798-1.042)	0.956 (0.815-1.121)		0.932 (0.788-1.102)
High	0.799***(0.685-0.933)	0.829*(0.675-1.019)		0.801**(0.643-0.997)
**Average community income**				
Low				

Lower-middle	0.948 (0.839-1.073)		1.009 (0.883-1.153)	1.035 (0.904-1.185)
Upper-middle	0.915 (0.797-1.051)		1.027 (0.871-1.212)	1.091 (0.919-1.297)
High	0.841**(0.721-0.981)		0.985 (0.813-1.194)	1.107 (0.903-1.357)
**Social group**				
SC/ST				
OBC	0.767***(0.692-0.849)	0.832***(0.744-0.93)	0.798***(0.714-0.892)	0.831***(0.743-0.929)
Others	0.638***(0.57-0.714)	0.722***(0.635-0.821)	0.65***(0.572-0.738)	0.721***(0.634-0.821)
**Religion**				
Hindu				
Muslim	0.727***(0.623-0.848)	0.785***(0.664-0.929)	0.883 (0.749-1.04)	0.782***(0.661-0.926)
Others	1.322***(1.136-1.539)	1.331***(1.132-1.564)	1.311***(1.116-1.541)	1.326***(1.127-1.559)
**Health Behaviour (consuming Alcohol/Tobacco)**				
No				
Yes	2.382***(2.183-2.601)	1.37***(1.238-1.516)	1.456***(1.316-1.61)	1.368***(1.236-1.515)
**Type of occupation**				
Not Working				
Professional & Admin/Manager	0.906 (0.693-1.183)	0.566***(0.423-0.758)	0.435***(0.327-0.578)	0.575***(0.428-0.772)
Clerical/Sales/Service	1.517***(1.284-1.793)	0.797**(0.664-0.957)	0.754***(0.628-0.905)	0.802**(0.667-0.964)
Farmers	1.356***(1.176-1.563)	0.554***(0.47-0.654)	0.609***(0.516-0.718)	0.555***(0.47-0.656)
Production	1.253***(1.096-1.433)	0.614***(0.528-0.714)	0.66***(0.568-0.768)	0.616***(0.53-0.716)
OCC Not Classified	1.11 (0.973-1.265)	0.573***(0.494-0.664)	0.587***(0.507-0.68)	0.574***(0.496-0.666)
**Region**				
North				
Central	0.984 (0.857-1.13)	0.957 (0.826-1.109)	0.936 (0.805-1.089)	0.972 (0.835-1.132)
East-Northeast	0.965 (0.84-1.109)	0.822***(0.712-0.95)	0.791***(0.682-0.918)	0.838**(0.721-0.973)
West	0.953 (0.816-1.113)	0.951 (0.809-1.117)	0.913 (0.776-1.074)	0.965 (0.819-1.137)
South	1.097 (0.961-1.252)	0.973 (0.844-1.121)	0.934 (0.807-1.081)	0.992 (0.854-1.151)
**Place of residence**				
Rural				
Urban	0.905**(0.821-0.998)	1.083 (0.959-1.222)	0.97 (0.864-1.089)	1.074 (0.95-1.216)
Random effects				
Level 2 (Community)		0.097(0.046-0.205)	0.104(0.052-0.210)	0.097(0.046-0.205)

Note: *p < = .05, **p < = .01, ***p < = .001, Confidence interval in parenthesis

Table S7. Multilevel model results: Odds ratios for female mid-age death between 2004-05 and 2011-12, with household income as the economic indicator.

**Variables**	Bivariate	Model 1 (Education effect)	Model 2 (Economic status effect)	Model 3 (Education vs Economic status effect)
**Morbidity**				
No Morbidity				
Cataract	4.469***(2.618-7.629)	2.085***(1.207-3.601)	2.211***(1.28-3.818)	2.096***(1.214-3.621)
Tuberculosis	5.789***(3.529-9.497)	4***(2.415-6.624)	4.301***(2.594-7.132)	4.031***(2.433-6.676)
High BP	1.771***(1.181-2.656)	1.148 (0.76-1.733)	1.105 (0.732-1.668)	1.138 (0.754-1.719)
Heart disease	4.472***(2.925-6.837)	3.015***(1.954-4.651)	2.958***(1.918-4.561)	3.015***(1.954-4.653)
Diabetes	5.379***(3.706-7.808)	3.673***(2.498-5.4)	3.338***(2.274-4.901)	3.695***(2.513-5.433)
Asthma	4.91***(3.177-7.589)	2.858***(1.828-4.466)	2.921***(1.867-4.568)	2.845***(1.819-4.449)
Others	3.064***(2.521-3.724)	2.361***(1.935-2.88)	2.354***(1.929-2.872)	2.338***(1.916-2.853)
**Age** squire (in log scale)	2.36***(2.171-2.566)	2.388***(2.131-2.676)	2.545***(2.277-2.843)	2.412***(2.151-2.704)
**Marital status**				
Marriage				
Unmarried	0.635***(0.536-0.752)	2.527***(2.01-3.176)	2.248***(1.795-2.815)	2.541***(2.021-3.194)
widowed	3.303***(2.813-3.879)	1.818***(1.53-2.16)	1.861***(1.566-2.212)	1.786***(1.502-2.123)
Separated	1.295 (0.938-1.787)	1.493**(1.077-2.07)	1.446**(1.043-2.005)	1.494**(1.077-2.073)
**Education level**				
No education				
Primary	0.588***(0.495-0.699)	0.748***(0.623-0.897)	0.93 (0.781-1.107)	0.755***(0.629-0.906)
Lower secondary	0.389***(0.335-0.452)	0.646***(0.538-0.775)	0.766***(0.659-0.891)	0.656***(0.546-0.788)
Upper secondary	0.314***(0.231-0.427)	0.58***(0.413-0.815)	0.751**(0.593-0.951)	0.588***(0.417-0.828)
Post-secondary & above	0.335***(0.241-0.465)	0.562***(0.386-0.818)	0.815 (0.626-1.061)	0.559***(0.381-0.819)
**Income group**				
Poorest				
Poorer	0.854*(0.721-1.012)			0.936 (0.786-1.114)
Middle	0.666***(0.581-0.763)			0.795***(0.683-0.924)
Rich	0.64***(0.517-0.792)			0.826 (0.651-1.047)
Richest	0.657***(0.523-0.826)			0.932 (0.713-1.219)
**Mean years of community schooling**				
Low				
Lower-middle	0.797***(0.694-0.917)	0.843**(0.726-0.977)		0.857**(0.737-0.997)
Upper-middle	0.678***(0.575-0.799)	0.743***(0.61-0.905)		0.766**(0.622-0.944)
High	0.528***(0.431-0.647)	0.574***(0.438-0.752)		0.567***(0.425-0.756)
**Average community income**				

Low				
Lower-middle	0.754***(0.652-0.871)		0.838**(0.716-0.98)	0.89 (0.759-1.042)
Upper-middle	0.672***(0.568-0.796)		0.818*(0.668-1.001)	0.942 (0.763-1.161)
High	0.652***(0.539-0.788)		0.825 (0.649-1.05)	1.041 (0.809-1.34)
**Social group**				
SC/ST				
OBC	0.725***(0.636-0.828)	0.73***(0.634-0.841)	0.698***(0.606-0.804)	0.739***(0.641-0.851)
Others	0.646***(0.56-0.746)	0.725***(0.616-0.855)	0.634***(0.539-0.745)	0.739***(0.626-0.872)
**Religion**				
Hindu				
Muslim	1.018 (0.855-1.212)	1.003 (0.829-1.214)	1.199*(0.996-1.443)	1.009 (0.833-1.221)
Others	0.985 (0.792-1.226)	0.975 (0.778-1.223)	0.896 (0.715-1.124)	0.967 (0.771-1.213)
**Health Behaviour (consuming Alcohol/Tobacco)**				
No				
Yes	2.566***(2.187-3.01)	1.542***(1.3-1.83)	1.571***(1.323-1.865)	1.516***(1.278-1.799)
**Type of occupation**				
Not Working				
Professional & Admin/Manager	0.491**(0.243-0.99)	0.541*(0.262-1.117)	0.419**(0.206-0.852)	0.533*(0.257-1.103)
Clerical/Sales/Service	1.024 (0.688-1.524)	0.622**(0.414-0.935)	0.629**(0.419-0.946)	0.62**(0.412-0.931)
Farmers	1.098 (0.925-1.304)	0.63***(0.519-0.764)	0.669***(0.551-0.812)	0.605***(0.499-0.735)
Production	1.042 (0.787-1.379)	0.646***(0.484-0.861)	0.692**(0.519-0.924)	0.636***(0.477-0.849)
OCC Not Classified	0.939 (0.821-1.074)	0.66***(0.569-0.765)	0.687***(0.593-0.797)	0.656***(0.566-0.761)
**Region**				
North				
Central	1.287***(1.084-1.528)	1.14 (0.951-1.365)	1.105 (0.917-1.332)	1.12 (0.93-1.349)
East-Northeast	1.199**(1.007-1.428)	1.004 (0.838-1.203)	0.89 (0.738-1.075)	0.971 (0.804-1.173)
West	0.855 (0.692-1.058)	0.897 (0.721-1.116)	0.778**(0.624-0.971)	0.879 (0.704-1.098)
South	1.145 (0.965-1.359)	1.063 (0.886-1.276)	0.886 (0.734-1.068)	1.046 (0.864-1.267)
**Place of residence**				
Rural				
Urban	0.847***(0.747-0.96)	0.981 (0.841-1.146)	0.854**(0.734-0.993)	1.013 (0.864-1.187)
Random effects				
Level 2 (Community)		0.028(0.001-1.252)	0.052(0.007-.415)	0.025(0.000-1.770)

Note: *p < = .05, **p < = .01, ***p < = .001, Confidence interval in parenthesis

Table S8. Multilevel model results: Odds ratios for male mid-age death between 2004-05 and 2011-12, with monthly consumption expenditure as the economic indicator.

**Variables**	Bivariate	Model 1 (Education effect)	Model 2 (Economic status effect)	Model 3 (Education vs Economic status effect)
**Morbidity**				
No Morbidity				
Cataract	5.212***(3.172-8.564)	2.138***(1.277-3.581)	2.263***(1.351-3.79)	2.144***(1.279-3.594)
Tuberculosis	5.06***(3.385-7.566)	3.035***(2.001-4.602)	3.291***(2.172-4.988)	3.039***(2.004-4.609)
High BP	2.964***(2.047-4.291)	1.728***(1.183-2.524)	1.702***(1.165-2.485)	1.749***(1.197-2.556)
Heart disease	5.433***(3.675-8.03)	3.457***(2.313-5.165)	3.452***(2.309-5.161)	3.477***(2.326-5.199)
Diabetes	6.071***(4.61-7.997)	3.486***(2.615-4.647)	3.406***(2.555-4.539)	3.517***(2.637-4.692)
Asthma	9.519***(6.98-12.982)	4.606***(3.331-6.37)	4.804***(3.475-6.641)	4.644***(3.358-6.423)
Others	3.999***(3.381-4.731)	2.74***(2.302-3.262)	2.892***(2.429-3.442)	2.761***(2.319-3.288)
**Age** squire (in log scale)	3.091***(2.884-3.313)	3.254***(2.952-3.585)	3.432***(3.119-3.777)	3.284***(2.979-3.621)
**Marital status**				
Marriage				
Unmarried	0.353***(0.314-0.398)	1.815***(1.52-2.166)	1.806***(1.514-2.153)	1.833***(1.536-2.189)
widowed	2.993***(2.368-3.783)	1.664***(1.3-2.13)	1.797***(1.404-2.299)	1.672***(1.306-2.141)
Separated	1.69**(1.097-2.603)	2.001***(1.276-3.136)	2.12***(1.352-3.324)	2.031***(1.295-3.184)
**Education level**				
No education				
Primary	0.798***(0.705-0.903)	0.92 (0.808-1.048)		0.929 (0.816-1.058)
Lower secondary	0.487***(0.438-0.542)	0.755***(0.667-0.855)		0.767***(0.677-0.869)
Upper secondary	0.31***(0.257-0.375)	0.535***(0.434-0.659)		0.548***(0.444-0.676)
Post-secondary & above	0.292***(0.238-0.359)	0.423***(0.333-0.537)		0.437***(0.343-0.557)
**Monthly consumption expenditure (MCE) group**				
Poorest				
Poorer	0.88*(0.766-1.012)		0.848**(0.732-0.984)	0.868*(0.748-1.006)
Middle	0.918 (0.826-1.021)		0.869**(0.766-0.985)	0.92 (0.811-1.044)
Rich	0.85**(0.725-0.998)		0.773***(0.641-0.932)	0.867 (0.718-1.046)
Richest	0.829**(0.698-0.985)		0.693***(0.563-0.854)	0.818*(0.662-1.011)
**Mean years of community schooling**				
Low				
Lower-middle	0.953 (0.847-1.073)	0.994 (0.875-1.129)		0.975 (0.855-1.112)
Upper-middle	0.912 (0.798-1.042)	0.956 (0.815-1.121)		0.916 (0.773-1.087)
High	0.799***(0.685-0.933)	0.829*(0.675-1.019)		0.768**(0.611-0.964)

**Average community MCE**				
Low				
Lower-middle	0.965 (0.856-1.088)		1.02 (0.892-1.166)	1.038 (0.906-1.191)
Upper-middle	0.976 (0.852-1.116)		1.056 (0.895-1.246)	1.122 (0.944-1.335)
High	0.922 (0.797-1.067)		1.037 (0.855-1.257)	1.209*(0.98-1.49)
**Social group**				
SC/ST				
OBC	0.767***(0.692-0.849)	0.832***(0.744-0.93)	0.808***(0.723-0.903)	0.838***(0.749-0.937)
Others	0.638***(0.57-0.714)	0.722***(0.635-0.821)	0.665***(0.584-0.756)	0.727***(0.638-0.829)
**Religion**				
Hindu				
Muslim	0.727***(0.623-0.848)	0.785***(0.664-0.929)	0.873 (0.74-1.03)	0.782***(0.661-0.925)
Others	1.322***(1.136-1.539)	1.331***(1.132-1.564)	1.306***(1.111-1.534)	1.333***(1.134-1.567)
**Health Behaviour (consuming Alcohol/Tobacco)**				
No				
Yes	2.382***(2.183-2.601)	1.37***(1.238-1.516)	1.463***(1.323-1.618)	1.369***(1.237-1.515)
**Type of occupation**				
Not Working				
Professional & Admin/Manager	0.906 (0.693-1.183)	0.566***(0.423-0.758)	0.426***(0.321-0.565)	0.573***(0.428-0.768)
Clerical/Sales/Service	1.517***(1.284-1.793)	0.797**(0.664-0.957)	0.745***(0.621-0.893)	0.799**(0.666-0.96)
Farmers	1.356***(1.176-1.563)	0.554***(0.47-0.654)	0.6***(0.509-0.708)	0.55***(0.466-0.649)
Production	1.253***(1.096-1.433)	0.614***(0.528-0.714)	0.654***(0.562-0.759)	0.611***(0.525-0.71)
OCC Not Classified	1.11 (0.973-1.265)	0.573***(0.494-0.664)	0.588***(0.508-0.682)	0.577***(0.497-0.668)
**Region**				
North				
Central	0.984 (0.857-1.13)	0.957 (0.826-1.109)	0.912 (0.781-1.064)	0.966 (0.827-1.128)
East-Northeast	0.965 (0.84-1.109)	0.822***(0.712-0.95)	0.777***(0.668-0.904)	0.841**(0.722-0.98)
West	0.953 (0.816-1.113)	0.951 (0.809-1.117)	0.906 (0.77-1.066)	0.972 (0.824-1.146)
South	1.097 (0.961-1.252)	0.973 (0.844-1.121)	0.942 (0.817-1.087)	0.994 (0.859-1.149)
**Place of residence**				
Rural				
Urban	0.905**(0.821-0.998)	1.083 (0.959-1.222)	0.976 (0.87-1.096)	1.079 (0.955-1.219)
Random effects				
Level 2 (Community)		.0974(0.0463-0.205)	0.107(0.054-0.212)	0.098(0.046-0.205)

Note: *p < = .05, **p < = .01, ***p < = .001, Confidence interval in parenthesis

Table S9. Multilevel model results: Odds ratios for female mid-age death between 2004-05 and 2011-12, with monthly consumption expenditure as the economic indicator.

**Variables**	Bivariate	Model 1 (Education effect)	Model 2 (Economic status effect)	Model 3 (Education vs Economic status effect)
**Morbidity**				
No Morbidity				
Cataract	4.469***(2.618-7.629)	2.085***(1.207-3.601)	2.184***(1.264-3.773)	2.064***(1.194-3.567)
Tuberculosis	5.789***(3.529-9.497)	4***(2.415-6.624)	4.397***(2.651-7.292)	4.066***(2.453-6.739)
High BP	1.771***(1.181-2.656)	1.148 (0.76-1.733)	1.16 (0.768-1.752)	1.171 (0.775-1.77)
Heart disease	4.472***(2.925-6.837)	3.015***(1.954-4.651)	3.072***(1.99-4.74)	3.035***(1.965-4.689)
Diabetes	5.379***(3.706-7.808)	3.673***(2.498-5.4)	3.487***(2.372-5.128)	3.806***(2.585-5.603)
Asthma	4.91***(3.177-7.589)	2.858***(1.828-4.466)	3.019***(1.93-4.722)	2.876***(1.838-4.499)
Others	3.064***(2.521-3.724)	2.361***(1.935-2.88)	2.488***(2.038-3.037)	2.412***(1.975-2.945)
**Age** squire (in log scale)	2.36***(2.171-2.566)	2.388***(2.131-2.676)	2.562***(2.294-2.862)	2.438***(2.174-2.734)
**Marital status**				
Marriage				
Unmarried	0.635***(0.536-0.752)	2.527***(2.01-3.176)	2.274***(1.817-2.847)	2.531***(2.014-3.181)
widowed	3.303***(2.813-3.879)	1.818***(1.53-2.16)	1.898***(1.598-2.254)	1.816***(1.528-2.157)
Separated	1.295 (0.938-1.787)	1.493**(1.077-2.07)	1.47**(1.06-2.039)	1.5**(1.081-2.08)
**Education level**				
No education				
Primary	0.588***(0.495-0.699)	0.748***(0.623-0.897)		0.764***(0.636-0.918)
Lower secondary	0.389***(0.335-0.452)	0.646***(0.538-0.775)		0.677***(0.563-0.814)
Upper secondary	0.314***(0.231-0.427)	0.58***(0.413-0.815)		0.63***(0.447-0.888)
Post-secondary & above	0.335***(0.241-0.465)	0.562***(0.386-0.818)		0.625**(0.426-0.917)
**Wealth group**				
Poorest				
Poorer	0.916 (0.771-1.087)		0.946 (0.792-1.13)	0.96 (0.803-1.147)
Middle	0.841**(0.736-0.962)		0.874*(0.753-1.015)	0.921 (0.793-1.071)
Rich	0.577***(0.458-0.728)		0.583***(0.453-0.749)	0.654***(0.508-0.843)
Richest	0.675***(0.535-0.852)		0.651***(0.502-0.843)	0.774*(0.594-1.009)
**Mean years of community schooling**				
Low				
Lower-middle	0.797***(0.694-0.917)	0.843**(0.726-0.977)		0.822**(0.705-0.959)
Upper-middle	0.678***(0.575-0.799)	0.743***(0.61-0.905)		0.715***(0.579-0.881)
High	0.528***(0.431-0.647)	0.574***(0.438-0.752)		0.547***(0.406-0.736)
**Average community MCE**				
Low				

Lower-middle	0.913 (0.79-1.055)		1.014 (0.864-1.19)	1.077 (0.916-1.267)
Upper-middle	0.821**(0.694-0.971)		1.013 (0.827-1.24)	1.186 (0.96-1.464)
High	0.691***(0.571-0.835)		0.923 (0.723-1.178)	1.239 (0.952-1.614)
**Social group**				
SC/ST				
OBC	0.725***(0.636-0.828)	0.73***(0.634-0.841)	0.702***(0.609-0.809)	0.739***(0.641-0.851)
Others	0.646***(0.56-0.746)	0.725***(0.616-0.855)	0.661***(0.561-0.778)	0.746***(0.631-0.881)
**Religion**				
Hindu				
Muslim	1.018 (0.855-1.212)	1.003 (0.829-1.214)	1.145 (0.95-1.38)	0.988 (0.816-1.196)
Others	0.985 (0.792-1.226)	0.975 (0.778-1.223)	0.9 (0.718-1.128)	0.981 (0.782-1.231)
**Health Behaviour (consuming Alcohol/Tobacco)**				
No				
Yes	2.566***(2.187-3.01)	1.542***(1.3-1.83)	1.582***(1.333-1.878)	1.533***(1.292-1.819)
**Type of occupation**				
Not Working				
Professional & Admin/Manager	0.491**(0.243-0.99)	0.541*(0.262-1.117)	0.45**(0.221-0.915)	0.549 (0.266-1.135)
Clerical/Sales/Service	1.024 (0.688-1.524)	0.622**(0.414-0.935)	0.623**(0.414-0.936)	0.616**(0.41-0.926)
Farmers	1.098 (0.925-1.304)	0.63***(0.519-0.764)	0.686***(0.565-0.832)	0.619***(0.51-0.752)
Production	1.042 (0.787-1.379)	0.646***(0.484-0.861)	0.696**(0.522-0.928)	0.635***(0.476-0.846)
OCC Not Classified	0.939 (0.821-1.074)	0.66***(0.569-0.765)	0.699***(0.603-0.81)	0.665***(0.573-0.77)
**Region**				
North				
Central	1.287***(1.084-1.528)	1.14 (0.951-1.365)	1.1 (0.911-1.329)	1.134 (0.939-1.369)
East-Northeast	1.199**(1.007-1.428)	1.004 (0.838-1.203)	0.9 (0.745-1.088)	1.003 (0.828-1.215)
West	0.855 (0.692-1.058)	0.897 (0.721-1.116)	0.779**(0.624-0.971)	0.899 (0.719-1.125)
South	1.145 (0.965-1.359)	1.063 (0.886-1.276)	0.914 (0.761-1.097)	1.062 (0.881-1.28)
**Place of residence**				
Rural				
Urban	0.847***(0.747-0.96)	0.981 (0.841-1.146)	0.846**(0.729-0.982)	0.987 (0.844-1.154)
Random effects				
Level 2 (Community)		0.028(0.001-1.252)	0.0538 (0.007-0.399)	0.031 (0.001-0.979)

Note: *p < = .05, **p < = .01, ***p < = .001, Confidence interval in parenthesis

Table S10. Multilevel model results: Odds ratios mid-age death between 2004-05 and 2011-12 for both male-female in rural area.

**Variables**	Bivariate	Model 1 (Education effect)	Model 2 (Economic status effect)	Model 3 (Education vs Economic status effect)
**Morbidity**				
No Morbidity				
Cataract	4.024***(2.597-6.237)	1.907***(1.214-2.996)	1.914***(1.218-3.008)	1.872***(1.19-2.945)
Tuberculosis	5.196***(3.654-7.388)	3.446***(2.397-4.952)	3.505***(2.437-5.041)	3.372***(2.345-4.849)
High BP	2.15***(1.479-3.126)	1.43*(0.977-2.094)	1.425*(0.973-2.086)	1.465*(1.001-2.146)
Heart disease	3.396***(2.221-5.193)	2.546***(1.651-3.924)	2.521***(1.636-3.885)	2.575***(1.67-3.968)
Diabetes	6.097***(4.409-8.43)	3.633***(2.596-5.085)	3.61***(2.579-5.053)	3.783***(2.7-5.299)
Asthma	7.773***(5.861-10.31)	4.278***(3.186-5.743)	4.242***(3.158-5.697)	4.203***(3.129-5.645)
Others	3.387***(2.917-3.931)	2.648***(2.271-3.088)	2.646***(2.269-3.086)	2.626***(2.251-3.063)
**Age** squire (in log scale)	2.72***(2.554-2.897)	2.797***(2.563-3.052)	2.797***(2.563-3.052)	2.847***(2.607-3.108)
**Sex**			2.962***(2.72-3.225)	
Male				
Female	0.589***(0.541-0.641)	0.55***(0.492-0.615)	0.601***(0.541-0.668)	0.554***(0.495-0.619)
**Marital status**				
Marriage				
Unmarried	0.489***(0.435-0.55)	2.018***(1.707-2.386)	1.923***(1.628-2.272)	2.006***(1.696-2.373)
widowed	2.363***(2.019-2.765)	1.542***(1.303-1.824)	1.544***(1.305-1.827)	1.51***(1.276-1.787)
Separated	1.014 (0.752-1.368)	1.511***(1.11-2.056)	1.498***(1.1-2.039)	1.501***(1.102-2.043)
**Education level**				
No education				
Primary	0.809***(0.722-0.907)	0.846***(0.75-0.955)		0.874**(0.774-0.987)
Lower secondary	0.534***(0.483-0.591)	0.729***(0.646-0.823)		0.778***(0.687-0.88)
Upper secondary	0.405***(0.329-0.499)	0.59***(0.47-0.741)		0.65***(0.516-0.819)
Post-secondary & above	0.431***(0.333-0.558)	0.556***(0.419-0.738)		0.631***(0.473-0.843)
**Wealth status group**				
Poorest				
Poorer	0.854**(0.754-0.966)		0.8***(0.704-0.909)	0.822***(0.723-0.935)
Middle	0.84***(0.762-0.927)		0.697***(0.618-0.787)	0.745***(0.658-0.842)
Rich	0.75***(0.63-0.894)		0.597***(0.481-0.742)	0.673***(0.539-0.839)
Richest	0.728***(0.599-0.886)		0.56***(0.432-0.724)	0.648***(0.497-0.844)
**Mean year of community schooling**				
Low				
Lower-middle	0.868***(0.784-0.962)	0.898*(0.806-1.002)		0.901*(0.802-1.011)
Upper-middle	0.795***(0.696-0.909)	0.829**(0.713-0.965)		0.817**(0.689-0.97)
High	0.809*(0.631-1.038)	0.682***(0.518-0.898)		0.643***(0.476-0.869)
**Average community wealth**				

Low				
Lower-middle	0.864***(0.777-0.961)		0.974 (0.868-1.093)	1.029 (0.912-1.159)
Upper-middle	0.766***(0.666-0.882)		0.961 (0.823-1.123)	1.103 (0.929-1.31)
High	0.965 (0.819-1.136)		1.161 (0.94-1.435)	1.383***(1.094-1.748)
**Social group**				
SC/ST				
OBC	0.776***(0.705-0.854)	0.813***(0.733-0.901)	0.82***(0.739-0.909)	0.835***(0.752-0.926)
Others	0.672***(0.601-0.751)	0.722***(0.637-0.818)	0.713***(0.629-0.809)	0.753***(0.663-0.855)
**Religion**				
Hindu				
Muslim	0.797***(0.679-0.936)	0.879 (0.741-1.042)	0.924 (0.779-1.096)	0.873 (0.736-1.036)
Others	1.191**(1.023-1.385)	1.179**(1.005-1.384)	1.137 (0.965-1.341)	1.159*(0.983-1.367)
**Health Behaviour (consuming Alcohol/Tobacco)**				
No				
Yes	2.557***(2.352-2.78)	1.438***(1.297-1.594)	1.443***(1.301-1.599)	1.417***(1.278-1.572)
**Type of occupation**				
Not Working				
Professional & Admin/Manager	0.885 (0.61-1.284)	0.535***(0.36-0.794)	0.459***(0.313-0.673)	0.548***(0.369-0.814)
Clerical/Sales/Service	1.818***(1.485-2.225)	0.907 (0.731-1.126)	0.858 (0.692-1.064)	0.914 (0.736-1.133)
Farmers	1.263***(1.122-1.423)	0.605***(0.528-0.693)	0.601***(0.524-0.689)	0.574***(0.5-0.658)
Production	1.268***(1.106-1.453)	0.607***(0.521-0.708)	0.598***(0.512-0.698)	0.586***(0.502-0.684)
OCC Not Classified	1.005 (0.9-1.121)	0.618***(0.549-0.696)	0.629***(0.559-0.709)	0.625***(0.555-0.704)
**Region**				
North				
Central	1.092 (0.954-1.249)	1.01 (0.876-1.165)	0.966 (0.827-1.129)	1.021 (0.873-1.195)
East-Northeast	1.083 (0.942-1.245)	0.891 (0.772-1.028)	0.823**(0.701-0.966)	0.908 (0.77-1.07)
West	0.997 (0.85-1.169)	1.024 (0.869-1.206)	0.957 (0.807-1.134)	1.058 (0.89-1.258)
South	1.16**(1.015-1.325)	1.062 (0.922-1.224)	0.959 (0.825-1.115)	1.104 (0.94-1.296)
Random effects				
Level 2 (Community)		0.101**(0.059-0.173)	0.113(0.069-0.184)	0.104(0.061-0.176)

Note: *p < = .05, **p < = .01, ***p < = .001, Confidence interval in parenthesis

Table S11. Multilevel model results: Odds ratios mid-age death between 2004-05 and 2011-12 for both male-female in urban area.

Variables	Bivariate	Model 1 (Education effect)	Model 2 (Economic status effect)	Model 3 (Education vs Economic status effect)
**Morbidity**				
No Morbidity				
Cataract	6.044***(3.168-11.53)	2.592***(1.321-5.087)	2.539***(1.291-4.993)	2.504***(1.275-4.92)
Tuberculosis	5.444***(2.779-10.663)	3.185***(1.578-6.426)	3.249***(1.608-6.564)	3.099***(1.534-6.261)
High BP	2.073***(1.395-3.08)	1.288 (0.858-1.933)	1.323 (0.881-1.986)	1.316 (0.877-1.976)
Heart disease	6.977***(4.711-10.332)	4.103***(2.729-6.167)	4.157***(2.766-6.248)	4.153***(2.762-6.243)
Diabetes	6.197***(4.568-8.407)	3.367***(2.45-4.627)	3.417***(2.485-4.7)	3.452***(2.509-4.751)
Asthma	5.634***(3.355-9.46)	2.899***(1.689-4.979)	2.917***(1.696-5.018)	2.843***(1.652-4.891)
Others	3.274***(2.572-4.167)	2.39***(1.862-3.066)	2.466***(1.921-3.165)	2.385***(1.858-3.062)
**Age** squire (in log scale)	3.007***(2.721-3.324)	3.066***(2.668-3.523)	3.279***(2.857-3.764)	3.172***(2.757-3.65)
**Sex**				
Male				
Female	0.548***(0.481-0.623)	0.423***(0.352-0.508)	0.468***(0.392-0.559)	0.423***(0.352-0.508)
**Marital status**				
Marriage				
Unmarried	0.421***(0.354-0.499)	1.847***(1.439-2.369)	1.787***(1.396-2.287)	1.879***(1.464-2.411)
widowed	2.612***(2.086-3.27)	1.767***(1.381-2.262)	1.857***(1.451-2.375)	1.741***(1.359-2.23)
Separated	1.224 (0.747-2.003)	1.933**(1.163-3.212)	1.826**(1.097-3.039)	1.867**(1.122-3.106)
**Education level**				
No education				
Primary	0.861 (0.711-1.044)	0.883 (0.72-1.083)	0.832 (0.64-1.081)	0.922 (0.75-1.132)
Lower secondary	0.554***(0.475-0.647)	0.719***(0.599-0.864)	0.651***(0.524-0.809)	0.782***(0.648-0.944)
Upper secondary	0.334***(0.259-0.429)	0.502***(0.378-0.668)	0.501***(0.381-0.659)	0.563***(0.42-0.754)
Post-secondary & above	0.32***(0.251-0.408)	0.401***(0.298-0.538)	0.482***(0.362-0.642)	0.454***(0.334-0.618)
**Wealth status**				
Poorest				
Poorer	0.972 (0.779-1.213)			0.848 (0.652-1.102)
Middle	0.729***(0.61-0.871)			0.701***(0.563-0.875)
Rich	0.638***(0.51-0.799)			0.585***(0.441-0.775)
Richest	0.63***(0.498-0.798)			0.626***(0.465-0.843)
**Mean year of community schooling**				
Low				
Lower-middle	1.035 (0.782-1.369)	1.084 (0.804-1.462)		1.058 (0.77-1.454)
Upper-middle	0.908 (0.689-1.196)	1 (0.736-1.359)		0.964 (0.679-1.368)
High	0.72**(0.547-0.948)	0.86 (0.62-1.191)		0.821 (0.554-1.217)

**Average community wealth**				
Low				
Lower-middle	0.965 (0.677-1.376)		1.095 (0.682-1.76)	1.104 (0.674-1.808)
Upper-middle	0.949 (0.667-1.351)		1.188 (0.747-1.888)	1.268 (0.773-2.08)
High	0.702*(0.489-1.008)		1.07 (0.664-1.724)	1.322 (0.77-2.271)
**Social group**				
SC/ST				
OBC	0.707***(0.603-0.83)	0.739***(0.621-0.879)	0.714***(0.6-0.85)	0.748***(0.628-0.891)
Others	0.611***(0.52-0.718)	0.692***(0.577-0.831)	0.634***(0.53-0.76)	0.711***(0.591-0.855)
**Religion**				
Hindu				
Muslim	0.919 (0.768-1.1)	0.887 (0.725-1.085)	0.98 (0.806-1.193)	0.881 (0.719-1.079)
Others	1.171 (0.921-1.488)	1.252*(0.976-1.606)	1.291**(1.005-1.66)	1.276*(0.993-1.641)
**Health Behaviour (consuming Alcohol/Tobacco)**				
No				
Yes	2.962***(2.594-3.381)	1.531***(1.305-1.797)	1.58***(1.347-1.854)	1.491***(1.269-1.751)
**Type of occupation**				
Not Working				
Professional & Admin/Manager	1.126 (0.819-1.548)	0.654**(0.456-0.937)	0.492***(0.349-0.693)	0.651**(0.454-0.933)
Clerical/Sales/Service	1.764***(1.448-2.149)	0.759**(0.605-0.953)	0.694***(0.554-0.87)	0.741***(0.591-0.93)
Farmers	1.334 (0.888-2.004)	0.437***(0.284-0.674)	0.423***(0.274-0.655)	0.4***(0.259-0.62)
Production	1.861***(1.58-2.194)	0.698***(0.569-0.856)	0.697***(0.567-0.856)	0.666***(0.542-0.818)
OCC Not Classified	1.326***(1.101-1.597)	0.632***(0.512-0.78)	0.629***(0.51-0.776)	0.629***(0.51-0.776)
**Region**				
North				
Central	1.075 (0.874-1.324)	1.057 (0.852-1.312)	0.987 (0.795-1.227)	1.026 (0.825-1.275)
East-Northeast	0.975 (0.8-1.189)	0.861 (0.703-1.054)	0.713***(0.575-0.884)	0.829 (0.663-1.037)
West	0.78**(0.62-0.982)	0.765**(0.605-0.967)	0.722***(0.569-0.917)	0.759**(0.598-0.963)
South	0.995 (0.821-1.207)	0.895 (0.729-1.098)	0.779**(0.628-0.966)	0.87 (0.695-1.088)
Random effects				
Level 2 (Community)		0.099(0.037-0.264)	0.115(0.048-0.272)	0.101(0.038-0.265)

Note: *p < = .05, **p < = .01, ***p < = .001, Confidence interval in parenthesis

Table S12. Mediation analysis results:

Part A: Decomposing effect of education and wealth effect on mid-age mortality by sex.

	Male	Female
**Effect of education**	Coefficient	Coefficient
Total effect	-0.241***(-0.296--0.186)	-0.224***(-0.305--0.143)
Direct effect	-0.188***(-0.245--0.131)	-0.195***(-0.277--0.113)
Indirect effect	-0.053***(-0.07--0.035)	-0.029***(-0.048--0.01)
% Indirect (mediated) effect	21.83%	13.04
**Effect of wealth**		
Total effect	-0.065**(-0.118--0.011)	-0.13***(-0.199--0.061)
direct effect	-0.024 (-0.08-0.031)	-0.088**(-0.159--0.018)
Indirect (Mediated) effect	-0.04***(-0.053--0.027)	-0.042***(-0.055--0.028)
% Indirect (mediated) effect	62.48	32.18

Note: Education is measured as years of schooling, and wealth is measured using an asset-based wealth index (asset count). Both variables were standardized (z-scores) to allow comparability of coefficients. *p ≤ .05, **p ≤ .01, ***p ≤ .001. 95% confidence intervals are shown in parentheses.

Part B: Contribution of individual mediators to the indirect effects of education and wealth on mid-age mortality

	Male			Female		
	Coefficient	Std	Contribution to mediated effect	Coefficient	Std	Contribution to mediated effect
**Effect of education**						
Morbidity	-0.005	0.001	2.09	0.000	0.001	0.04
Marriage	-0.004	0.001	1.53	-0.001	0.001	0.4
Health behaviour	-0.015	0.004	6.21	-0.004	0.001	1.81
Caste	-0.011	0.002	4.65	-0.013	0.003	5.85
Occupation	-0.016	0.007	6.66	-0.009	0.009	4.15
Religion	-0.002	0.004	0.69	-0.002	0.002	0.81
**Effect of wealth**						
Morbidity	0.002	0.001	-2.58	0.001	0.001	-0.94
Marriage	-0.002	0.001	3.01	-0.008	0.001	6.21
Health behaviour	-0.013	0.003	20	-0.012	0.003	9.5
Caste	-0.025	0.005	39.04	-0.025	0.006	19.11
Occupation	-0.003	0.001	5.36	0.000	0.000	0.3
Religion	0.002	0.003	-2.35	0.003	0.003	-2

Table S13. Mediation analysis results:

Part A: decomposing of education and wealth effects on mid-age mortality by sex, considering each as a mediator for the other

	Male	Female
**Effect of education**	Coefficient	Coefficient
Total effect	-0.277***(-0.323--0.231)	-0.301***(-0.37--0.231)
direct effect	-0.188***(-0.245--0.131)	-0.195***(-0.277--0.113)
Indirect (Mediated) effect	-0.089***(-0.124--0.054)	-0.106***(-0.151--0.061)
% Indirect (mediated) effect	32.09	35.33
**Effect of Wealth**		
Total effect	-0.186***(-0.231--0.141)	-0.247***(-0.307--0.188)
direct effect	-0.024 (-0.08-0.031)	-0.088**(-0.159--0.018)
Indirect (Mediated) effect	-0.161***(-0.192--0.131)	-0.159***(-0.203--0.115)
% Indirect (mediated) effect	86.95	64.36

Note: Education is measured as years of schooling, and wealth is measured using an asset-based wealth index (asset count). Both variables were standardized (z-scores) to allow comparability of coefficients. *p ≤ .05, **p ≤ .01, ***p ≤ .001. 95% confidence intervals are shown in parentheses.

Part B. Contribution of individual mediators to the indirect effects of education and wealth on mid-age mortality.

	Male			Female		
	Coefficient	Std. err.	Contribution to mediated effect	Coefficient	Std. err.	Contribution to mediated effect
**Effect of Education**						
Wealth	-0.014	0.016	4.93	-0.052	0.021	17.38
Morbidity	-0.004	0.001	1.48	0.001	0.001	-0.21
Marriage	-0.005	0.001	1.73	-0.006	0.001	1.89
Health behaviour	-0.022	0.006	8.02	-0.011	0.003	3.78
Caste	-0.025	0.005	9.17	-0.028	0.006	9.24
Occupation	-0.018	0.007	6.49	-0.010	0.009	3.16
Religion	-0.001	0.002	0.29	0.000	0.000	0.09
**Effect of wealth**						
Education	-0.095	0.015	50.98	-0.102	0.022	41.26
Morbidity	-0.001	0.001	0.47	0.001	0.001	-0.47
Marriage	-0.004	0.001	2.05	-0.009	0.001	3.45
Health behaviour	-0.020	0.005	11	-0.014	0.003	5.85
Caste	-0.031	0.006	16.61	-0.032	0.007	12.82
Occupation	-0.012	0.005	6.21	-0.005	0.005	2.13
Religion	0.001	0.001	-0.37	0.002	0.002	-0.67

Table S14. Multilevel model results: Odds ratios for male prime-age death between 2004-05 and 2011-12 adjustment for attrition.

Variables	Bivariate	Model 1 (Education effect)	Model 2 (Economic status effect)	Model 3 (Education vs Economic status effect)
**Morbidity**				
No Morbidity				
Cataract	4.991***(3.045-8.182)	2.099***(1.257-3.505)	2.103***(1.26-3.51)	2.058***(1.232-3.439)
Tuberculosis	4.742***(3.179-7.074)	2.724***(1.802-4.118)	2.809***(1.857-4.248)	2.669***(1.764-4.037)
High BP	2.897***(2.004-4.189)	1.707***(1.173-2.486)	1.694***(1.161-2.472)	1.744***(1.195-2.545)
Heart disease	5.117***(3.403-7.696)	3.236***(2.119-4.942)	3.189***(2.092-4.863)	3.263***(2.139-4.977)
Diabetes	5.567***(4.272-7.253)	3.271***(2.478-4.319)	3.272***(2.478-4.321)	3.361***(2.545-4.439)
Asthma	8.885***(6.534-12.083)	4.265***(3.094-5.879)	4.321***(3.135-5.956)	4.217***(3.059-5.816)
Others	3.896***(3.301-4.599)	2.629***(2.215-3.12)	2.659***(2.239-3.157)	2.599***(2.189-3.086)
**Age** squire (in log scale)	3.071***(2.855-3.303)	3.339***(3.036-3.671)	3.523***(3.211-3.865)	3.398***(3.089-3.738)
**Marital status**				
Marriage				
Unmarried	0.363***(0.317-0.414)	1.887***(1.577-2.259)	1.84***(1.54-2.199)	1.895***(1.583-2.269)
widowed	3.007***(2.362-3.829)	1.623***(1.257-2.095)	1.676***(1.3-2.162)	1.591***(1.232-2.055)
Separated	1.614**(1.046-2.491)	1.825***(1.162-2.867)	1.813***(1.153-2.851)	1.797**(1.144-2.825)
**Education level**				
No education				
Primary	0.797***(0.7-0.908)	0.931 (0.813-1.066)		0.954 (0.833-1.093)
Lower secondary	0.488***(0.437-0.544)	0.757***(0.667-0.86)		0.801***(0.703-0.911)
Upper secondary	0.316***(0.262-0.382)	0.544***(0.44-0.671)		0.59***(0.479-0.728)
Post-secondary & above	0.294***(0.243-0.357)	0.429***(0.341-0.539)		0.47***(0.371-0.594)
**Wealth group**				
Poorest				
Poorer	0.774***(0.67-0.894)		0.789***(0.671-0.927)	0.817**(0.695-0.96)
Middle	0.726***(0.656-0.804)		0.712***(0.62-0.817)	0.771***(0.67-0.886)
Rich	0.609***(0.512-0.725)		0.569***(0.458-0.707)	0.678***(0.543-0.847)
Richest	0.609***(0.496-0.746)		0.546***(0.416-0.716)	0.707**(0.534-0.935)
**Mean year of community schooling**				
Low				
Lower-middle	0.944 (0.839-1.063)	0.992 (0.871-1.13)		0.974 (0.849-1.118)
Upper-middle	0.885*(0.776-1.009)	0.951 (0.805-1.123)		0.913 (0.755-1.105)
High	0.752***(0.648-0.874)	0.817**(0.67-0.997)		0.766**(0.606-0.968)
**Average community wealth quintile**				
Low				
Lower-middle	0.949 (0.84-1.073)	0***(0-0)	1.081 (0.946-1.236)	1.106 (0.961-1.273)
Upper-middle	0.892*(0.782-1.017)	0***(0-0)	1.119 (0.951-1.316)	1.191*(0.995-1.427)
High	0.835***(0.733-0.951)	0***(0-0)	1.104 (0.904-1.349)	1.297**(1.024-1.642)

**Social group**				
SC/ST				
OBC	0.762***(0.688-0.843)	0.828***(0.742-0.924)	0.821***(0.736-0.916)	0.84***(0.753-0.938)
Others	0.633***(0.567-0.707)	0.72***(0.636-0.816)	0.685***(0.605-0.775)	0.739***(0.652-0.838)
**Religion**				
Hindu				
Muslim	0.725***(0.619-0.849)	0.801**(0.676-0.949)	0.874 (0.739-1.034)	0.799***(0.674-0.948)
Others	1.271***(1.096-1.474)	1.291***(1.103-1.511)	1.319***(1.124-1.548)	1.297***(1.104-1.523)
**Health Behaviour (consuming Alcohol/Tobacoo)**				
No				
Yes	2.333***(2.131-2.555)	1.337***(1.209-1.478)	1.378***(1.245-1.525)	1.316***(1.189-1.457)
**Type of occupation**				
Not Working				
Professional & Admin/Manager	0.914 (0.685-1.22)	0.578***(0.425-0.788)	0.444***(0.328-0.6)	0.587***(0.43-0.801)
Clerical/Sales/Service	1.46***(1.212-1.759)	0.776**(0.639-0.942)	0.725***(0.598-0.879)	0.778**(0.641-0.945)
Farmers	1.405***(1.201-1.643)	0.565***(0.475-0.671)	0.574***(0.483-0.683)	0.542***(0.457-0.644)
Production	1.265***(1.095-1.462)	0.619***(0.529-0.723)	0.628***(0.539-0.733)	0.601***(0.515-0.701)
OCC Not Classified	1.14*(0.995-1.305)	0.583***(0.502-0.677)	0.596***(0.513-0.692)	0.589***(0.507-0.684)
Other	1.33 (0.319-5.549)	0.656 (0.153-2.82)	0.637 (0.148-2.732)	0.659 (0.153-2.833)
**Region**				
North				
Central	1.014 (0.885-1.162)	0.968 (0.837-1.119)	0.902 (0.776-1.048)	0.964 (0.827-1.122)
East-Northeast	0.951 (0.831-1.088)	0.811***(0.705-0.932)	0.728***(0.625-0.848)	0.816**(0.695-0.958)
West	0.97 (0.832-1.13)	0.958 (0.816-1.124)	0.898 (0.765-1.055)	0.965 (0.819-1.137)
South	1.06 (0.933-1.205)	0.941 (0.821-1.08)	0.866*(0.75-1)	0.958 (0.824-1.114)
**Place of residence**				
Rural				
Urban	0.857***(0.776-0.946)	1.038 (0.919-1.171)	0.989 (0.864-1.133)	1.038 (0.909-1.187)
Random effects				
Level 2 (Community)		0.087(0.016-0.158)	0.097(0.026-0.168)	0.087(0.016-0.0159)

Note: *p < = .05, **p < = .01, ***p < = .001, Confidence interval in parenthesis

Table S15. Multilevel model results: Odds ratios of female prime-age death between 2004-05 and 2011-12 adjustment for attrition.

Variables	Bivariate	Model 1 (Education effect)	Model 2 (Economic status effect)	Model 3 (Education vs Economic status effect)
**Morbidity**				
No Morbidity				
Cataract	4.575***(2.745-7.626)	2.069***(1.224-3.497)	2.086***(1.234-3.528)	2.048***(1.21-3.464)
Tuberculosis	5.421***(3.31-8.88)	3.645***(2.202-6.032)	3.782***(2.282-6.27)	3.607***(2.178-5.974)
High BP	1.991***(1.332-2.977)	1.245 (0.824-1.881)	1.248 (0.822-1.893)	1.261 (0.833-1.909)
Heart disease	4.418***(2.854-6.838)	2.851***(1.817-4.472)	2.834***(1.812-4.432)	2.878***(1.837-4.507)
Diabetes	5.364***(3.626-7.936)	3.359***(2.246-5.024)	3.285***(2.194-4.917)	3.442***(2.302-5.147)
Asthma	4.662***(3.042-7.144)	2.664***(1.716-4.136)	2.695***(1.736-4.186)	2.633***(1.696-4.088)
Others	2.917***(2.402-3.544)	2.186***(1.791-2.668)	2.198***(1.8-2.682)	2.181***(1.787-2.662)
**Age** squire (in log scale)	2.464***(2.27-2.674)	2.56***(2.297-2.852)	2.74***(2.462-3.049)	2.606***(2.334-2.91)
**Marital status**				
Marriage				
Unmarried	0.619***(0.526-0.728)	2.625***(2.101-3.282)	2.361***(1.904-2.927)	2.638***(2.112-3.294)
widowed	3.175***(2.701-3.733)	1.696***(1.426-2.017)	1.734***(1.455-2.067)	1.679***(1.409-1.999)
Separated	1.248 (0.902-1.726)	1.434**(1.031-1.994)	1.407**(1.012-1.957)	1.434**(1.032-1.992)
**Education level**				
No education				
Primary	0.618***(0.514-0.744)	0.769***(0.633-0.934)		0.797**(0.653-0.972)
Lower secondary	0.407***(0.354-0.467)	0.658***(0.556-0.779)		0.696***(0.585-0.829)
Upper secondary	0.31***(0.228-0.42)	0.55***(0.392-0.773)		0.59***(0.42-0.83)
Post-secondary & above	0.337***(0.235-0.485)	0.536***(0.357-0.803)		0.567***(0.382-0.842)
**Wealth group**				
Poorest				
Poorer	0.702***(0.589-0.838)	0***(0-0)	0.755***(0.623-0.914)	0.775***(0.639-0.94)
Middle	0.655***(0.577-0.744)	0***(0-0)	0.707***(0.599-0.836)	0.764***(0.644-0.907)
Rich	0.552***(0.426-0.716)	0***(0-0)	0.563***(0.409-0.775)	0.659**(0.477-0.91)
Richest	0.603***(0.461-0.789)	0***(0-0)	0.622***(0.447-0.866)	0.785 (0.565-1.09)
**Mean year of community schooling**				
Low				
Lower-middle	0.807***(0.701-0.929)	0.843**(0.727-0.978)		0.875 (0.745-1.028)
Upper-middle	0.708***(0.603-0.83)	0.762***(0.632-0.919)		0.783**(0.633-0.968)
High	0.593***(0.488-0.722)	0.632***(0.489-0.816)		0.606***(0.449-0.819)
**Average community wealth quintile**				
Low				
Lower-middle	0.78***(0.671-0.906)	0***(0-0)	0.87 (0.736-1.029)	0.936 (0.786-1.114)
Upper-middle	0.697***(0.596-0.815)	0***(0-0)	0.851 (0.69-1.049)	0.997 (0.795-1.25)
High	0.668***(0.565-0.79)	0***(0-0)	0.852 (0.656-1.106)	1.178 (0.882-1.573)

**Social group**				
SC/ST				
OBC	0.731***(0.643-0.83)	0.731***(0.637-0.838)	0.727***(0.635-0.834)	0.751***(0.655-0.861)
Others	0.647***(0.559-0.75)	0.715***(0.605-0.846)	0.662***(0.561-0.781)	0.738***(0.623-0.874)
**Religion**				
Hindu				
Muslim	1.001 (0.844-1.187)	1.011 (0.841-1.214)	1.131 (0.945-1.355)	1.01 (0.84-1.213)
Others	1.002 (0.805-1.246)	0.98 (0.78-1.23)	0.954 (0.754-1.206)	0.972 (0.768-1.231)
**Health Behaviour (consuming Alcohol/Tobacco)**				
No				
Yes	2.468***(2.098-2.904)	1.464***(1.23-1.743)	1.481***(1.242-1.767)	1.436***(1.206-1.709)
**Type of occupation**				
Not Working				
Professional & Admin/Manager	0.524*(0.27-1.019)	0.565*(0.287-1.11)	0.445**(0.227-0.875)	0.563*(0.286-1.106)
Clerical/Sales/Service	0.999 (0.672-1.486)	0.61**(0.404-0.922)	0.588**(0.389-0.889)	0.594**(0.393-0.899)
Farmers	1.093 (0.926-1.291)	0.636***(0.525-0.77)	0.624***(0.514-0.757)	0.593***(0.489-0.72)
Production	1.027 (0.776-1.358)	0.654***(0.493-0.868)	0.653***(0.49-0.868)	0.627***(0.471-0.833)
OCC Not Classified	0.925 (0.81-1.057)	0.653***(0.564-0.755)	0.664***(0.573-0.77)	0.651***(0.562-0.755)
Other	2.582 (0.331-20.144)	1.481 (0.176-12.458)	1.443 (0.17-12.219)	1.536 (0.184-12.791)
**Region**				
North				
Central	1.257***(1.063-1.488)	1.118 (0.936-1.336)	1.053 (0.878-1.262)	1.114 (0.928-1.338)
East-Northeast	1.217**(1.025-1.446)	1.032 (0.866-1.229)	0.87 (0.724-1.046)	1.026 (0.848-1.242)
West	0.87 (0.69-1.098)	0.898 (0.703-1.146)	0.813*(0.641-1.03)	0.917 (0.719-1.17)
South	1.173*(0.988-1.392)	1.082 (0.899-1.301)	0.901 (0.752-1.08)	1.101 (0.904-1.341)
**Place of residence**				
Rural				
Urban	0.875**(0.773-0.991)	1.009 (0.864-1.178)	0.964 (0.821-1.133)	1.025 (0.871-1.206)
Random effects				
Level 2 (Community)		0.036 (-0.066-0.137)	0.053(-.056-0.161)	0.035(-0.066-0.137)

Note: *p < = .05, **p < = .01, ***p < = .001, Confidence interval in parenthesis

SI References

1. United Nations, "World Population Prospects: The 2019 Revision" (Department of Economic and Social Affairs, Population Division, 2019).

2. D. Barik, S. Desai, R. Vanneman, Economic status and adult mortality in India: is the relationship sensitive to choice of indicators? *World Dev.* **103**, 176–187 (2018).

3. N. Saikia, F. Ram, Determinants of adult mortality in India. *Asian Popul. Stud.* **6**, 153–171 (2010).

4. A. Gupta, N. Sudharsanan, Large and persistent life expectancy disparities between India's social groups. *Popul. Dev. Rev.* **48**, 863–882 (2022).

5. M. Asaria, *et al.*, Socioeconomic inequality in life expectancy in India. *BMJ Glob. Health* **4**, e001445 (2019).

6. T. Dyson, Excess male mortality in India. *Econ. Polit. Wkly.* 422–426 (1984).

7. N. Saikia, A. Singh, F. Ram, Adult male mortality in India: an application of the widowhood method. *Asian Popul. Stud.* **9**, 244–263 (2013).

8. U. Ram, *et al.*, Age-specific and sex-specific adult mortality risk in India in 2014: analysis of 0· 27 million nationally surveyed deaths and demographic estimates from 597 districts. *Lancet Glob. Health* **3**, e767–e775 (2015).

9. N. Krishnaji, K. James, Gender differentials in adult mortality: with notes on rural-urban contrasts. *Econ. Polit. Wkly.* 4633–4637 (2002).

10. A. D. Lopez, G. Caselli, T. Valkonen, *Adult mortality in developed countries: from description to explanation* (Oxford University Press, 1995).

11. S. Desai, R. Vanneman, N. D. National Council of Applied Economic Research, India Human Development Survey Panel (IHDS, IHDS-II), 2005, 2011-2012. Inter-university Consortium for Political and Social Research. https://doi.org/10.3886/ICPSR37382.v1. Deposited 19 November 2019.

12. United Nations, World Population Prospects 2024, Online Edition. Deposited 2024.

13. K. A. Bollen, J. L. Glanville, G. Stecklov, Socioeconomic status and class in studies of fertility and health in developing countries. *Annu. Rev. Sociol.* **27**, 153–185 (2001).

14. D. Filmer, L. H. Pritchett, Estimating wealth effects without expenditure data-or tears: An application to educational enrollments in states of India. *Demography* **38**, 115–132 (2001).

15. D. Barik, S. Desai, R. Vanneman, Economic Status and Adult Mortality in India: Is the Relationship Sensitive to Choice of Indicators? *World Dev.* **103**, 176–187 (2018).

16. E. R. Pamuk, R. Fuchs, W. Lutz, Comparing relative effects of education and economic resources on infant mortality in developing countries. *Popul. Dev. Rev.* **37**, 637–664 (2011).

17. Ø. Kravdal, Child mortality in India: The community-level effect of education. *Popul. Stud.* **58**, 177–192 (2004).

18. D. B. Rubin, Inference and missing data. *Biometrika* **63**, 581–592 (1976).

19. J. P. Reiter, T. E. Raghunathan, The multiple adaptations of multiple imputation. *J. Am. Stat. Assoc.* **102**, 1462–1471 (2007).

20. J. B. Carlin, J. C. Galati, P. Royston, A new framework for managing and analyzing multiply imputed data in Stata. *Stata J.* **8**, 49–67 (2008).

21. P. Royston, J. B. Carlin, I. R. White, Multiple imputation of missing values: new features for mim. *Stata J.* **9**, 252–264 (2009).

22. I. R. White, P. Royston, A. M. Wood, Multiple imputation using chained equations: issues and guidance for practice. *Stat. Med.* **30**, 377–399 (2011).

23. J. R. Carpenter, J. H. Roger, M. G. Kenward, Analysis of longitudinal trials with protocol deviation: a framework for relevant, accessible assumptions, and inference via multiple imputation. *J. Biopharm. Stat.* **23**, 1352–1371 (2013).

24. D. R. Johnson, R. Young, Toward best practices in analyzing datasets with missing data: Comparisons and recommendations. *J. Marriage Fam.* **73**, 926–945 (2011).

25. R. Young, D. R. Johnson, Imputing the Missing Y's: Implications for Survey Producers and Survey users: Proceedings of the AAPOR Conference Abstracts in (2010), pp. 6242–6248.
